# The role of fibroblast growth factor 18 in cancers: functions and signaling pathways

**DOI:** 10.3389/fonc.2023.1124520

**Published:** 2023-05-09

**Authors:** Yiming Zhou, Sizheng Sun, Tao Ling, Yongzhen Chen, Rongzhong Zhou, Qiang You

**Affiliations:** ^1^ Department of Biotherapy, Medical Center for Digestive Diseases, Second Affiliated Hospital, Nanjing Medical University, Nanjing, China; ^2^ Department of General Surgery, The Second Affiliated Hospital of Nanjing Medical University, Nanjing, Jiangsu, China; ^3^ Second Affiliated Hospital, Nanjing Medical University, Nanjing, China; ^4^ Department of Ophthalmology, Zaoyang First People’s Hosipital, Zaoyang, China

**Keywords:** FGF18, (FGFR) fibroblast growth factor receptor, cancer, gene expression, pathway

## Abstract

Fibroblast growth factor 18(FGF18) is a member of the fibroblast growth factor family (FGFs). FGF18 is a class of bioactive substances that can conduct biological signals, regulate cell growth, participate in tissue repair and other functions, and can promote the occurrence and development of different types of malignant tumors through various mechanisms. In this review, we focus on recent studies of FGF18 in the diagnosis, treatment, and prognosis of tumors in digestive, reproductive, urinary, respiratory, motor, and pediatric systems. These findings suggest that FGF18 may play an increasingly important role in the clinical evaluation of these malignancies. Overall, FGF18 can function as an important oncogene at different gene and protein levels, and can be used as a potential new therapeutic target and prognostic biomarker for these tumors.

## Introduction

Cancer is one of the biggest threats to human health, and how to prevent, screen and treat cancer at an early stage is a major problem at present (1–5). Therefore, current research focuses on the exploration of oncogenes, such as the activation of proto-oncogenes and the inactivation of tumor suppressor genes (6–9), among which many cytokines, including fibroblast growth factor 18, are believed to be involved in the occurrence and development of tumors (10–13).

Fibroblast growth factor 18 is a member of the fibroblast growth factor family (FGFs). FGFs is a class of bioactive substances that can promote fibroblast growth initially extracted from tissue extracts of brain and pituitary (14). There are more FGF family members classified according to different criteria, and 22 FGFs and 4 FGFR species have been identified in mammals to date (15, 16). According to their molecular structure, 22 FGFs species could be divided into 7 subgroups, among which, FGF8, FGF17 and FGF18 belonged to the FGF8 family subfamily (11, 14, 17). Fibroblast growth factors (FGF) and their receptors (FGFRs) drive key developmental signaling pathways that are responsible for many functions, including cell proliferation, survival, and migration (18–20). Thus, both FGFs and FGFR frequently play a role in cancer cell progression and have been shown to be carcinogenic in many cancers (19, 21–23).

In 1998, Japanese scientist Ohbayashi (24) isolated FGF18 from mouse embryos for the first time. FGF18 is a pleiotropic growth factor that stimulates the proliferation of interstitial and epithelial cells and tissues in a variety of organs, such as lung, liver, stomach, colorectum, brain, ovary, and embryo. It is also involved in cortical neuronal activity, cartilage and osteogenic development, and hair growth (25–28). FGF18 performs cell signal transduction by binding to the FGF receptor, and the function of FGF18 depends on the expression of FGFR in cell tissues (29, 30).

The function of FGF18 is not limited to the metabolism and development of tissues and organs. FGF18 is a potent mitogen for a variety of cells and plays an important role in directing cancers in organs or systems such as digestion and reproduction (31). For example, Shimokawa et al. (32) further emphasized the correlation between FGF18 and digestive system balance, and proposed that increased FGF18 expression may lead to the occurrence of colorectal tumors. In 2013, Wei’s team predicted that overexpression of FGF18 was an independent marker for poor prognosis of ovarian cancer, and determined that FGF18 was a serum biomarker of ovarian cancer, which was significantly correlated with the prognosis and progression of ovarian cancer (33). In addition, the continuous discovery of FGF18 in different organs or systems is of great significance in guiding tumor progression, which indicates that FGF18 can be used as an oncogene reference for the progression of multiple systemic tumors (10, 31–36).

Although only a few functions of FGF18 in tumors have been reported, FGF18 has become an essential regulatory factor in tumor progression and is involved in controlling various physiological processes of tumors in multiple systems. To date, the exact molecular mechanism of FGF18 in cancer remains unclear. Here, we review the regulatory mechanisms of FGF18 in different tumors, and further studies are expected to provide new therapeutic targets for these diseases.

## The role of FGF18 in cancer

### Gastric cancer

Gastric cancer (GC) is the second most common cancer in the world in terms of deaths and the fifth most common cancer in terms of incidence (37). These are major risk factors for the development of gastric cancer through dietary disorders, such as smoked or preserved foods, and smoking and alcohol consumption (38, 39). For most countries, the diagnosis is usually too late for radical treatment due to the lack of a good system in terms of diagnostic and screening methods (40–42). Although the same therapies can effectively improve overall survival (OS) in these patients, understanding the molecular mechanisms of their pathogenesis and identifying new therapeutic targets are still urgently needed.

In terms of its clinical relevance, FGF18 is a valid diagnostic indicator in GC (43, 44). In 2019, Austrian scientist Gerd conducted a preliminary analysis of FGF18 mRNA expression data of 87 patients with esophagogastric junction adenocarcinoma(AEG) in the Cancer Genome Atlas (TCGA) database, predicting that FGF18 has a high level of expression in AEG. The use of immunohistochemistry(IHC) to detect FGF18 protein levels in diagnostic biopsies and postoperative specimens from 155 patients was also studied, with high levels of FGF18 expression detected in samples from 49 patients (31.6%). Moreover, the established multivariate Cox proportional hazard regression model showed that overexpression of FGF18 was significantly associated with longer survival in AEG patients (45). In a cohort study of 75 patients including GC and peptic ulcer (PU), the mean value of FGF18 in GC was significantly higher than that of normal people, and the level of FGF18 in GC patients was 2.4 times higher than that in gastric ulcer (GU) or PU patients, potentially regulating the progression of GU to GC (46, 47). In conclusion, FGF18 is a promising key biomarker for GC or AEG prognostic factors, as well as an important target for new anticancer therapies.

FGF18 is one of the representative inflammatory markers (48), and in further exploration of the progression of Helicobacter pylori-induced GU or PU to GC. RNAseq analysis and Medline database showed that FGF18 was significantly increased in gastric epithelial cells after H. pylori infection (49). Egyptian scientist Mona Schaalan is exploring long non-coding RNA H19 (lncRNA H19) and related microRNA (miRNA): When miRNA 200c/miRNA 139 and miRNA-204/miRNA-182 were used as novel predictors of therapeutic response in HP induced GU or PU progression to GC, it was found that lncRNA H19 (50) levels were significantly increased in GC patients compared with GU subjects. The associated downstream targets miRNA 200c/miRNA 139 and miRNA-204/miRNA-182 were significantly down-regulated, and lncRNA H19 was also significantly correlated with FGF18, an important mediator in its regulation.Therefore, to some extent, FGF18 may be an important regulator in the process of potential diagnostic biomarkers for early GC diagnosis (46, 47).

The carcinogenic activity of FGF18 is closely related to various GC pathways and plays a prominent driving role in the occurrence of GC, with functions such as gene amplification or somatic mutation (44). In 2019, Zhang et al. explored the FGF mRNA profile in 10 GC cell lines by microarray analysis and found that the mRNA of FGF18 exhibited significantly high levels of expression. Human recombinant FGF18 protein and FGF18 conditioned medium were used to stimulate AGS and other gastric cancer cell lines. Western blotting (WB) and immunofluorescence (IF) experiments verified that FGF18 promoted cell growth by activating SMAD2/3 and inhibiting ATM signaling in an autocrine manner. In addition, ERK-MAPK signaling was also activated and accelerated tumor tissue growth, ultimately confirming FGF18 as a direct target of tumor suppressor miR-590-5p in inhibiting GC cell growth (43). Therefore, in 2020, Zhang et al. found that after overexpression of FGF18, FGFR2 expression could be enhanced, and then the expression of F-actin and YAP1 target was also enhanced, and nuclear accumulation of YAP1 occurred. These effects were also confirmed with FGFR2 depletion, knockdown, or the use of FGFR inhibitor AZD4547 (51, 52). In addition, with the inhibition of FGF18, the expression of c-Jun (effector of MAPK signaling) was also decreased. Through clinical data analysis and sample studies, the clinical prognosis of GC was unfavorable with the activation of the FGF18-FGFR2-c-Jun-YAP1 axis (53).

In conclusion, FGF18 affects the development of GC cells at different levels, and can be used as a novel prognostic marker and therapeutic target for GC. However, the molecular network structure of FGF18 in gastric cancer still needs to be further explored.

### Colorectal cancer

Today colorectal cancer(CRC) is the third leading cause of cancer mortality (54), and its incidence is age-related and increases with age (55, 56). Unhealthy diet and lifestyle habits, collective metabolic disorders, and genetics are all associated with the development of CRC (57). Although the literature provides a more systematic framework for the cancer characteristics of CRC, the understanding of its effective therapeutic advances remains much less well understood (58).

Expression of FGF18 in CRC and its effect on clinical prognosis. Masuko, a Japanese scientist, used the Basic Local Alignment Search Tool(BLAST) program to search for human EST(Expressed Sequence Tag) derived from FGF 18 mRNA, Analysis revealed that FGF18 mRNA can be expressed in CRC (11). The expression of FGF18 gene in CRC was significantly increased by genome-wide cDNA microarray analysis of CRC clinical samples (32). In 2020, Xu et al. used univariate Cox regression analysis to assess the prognostic risk of stage IV CRC patients and predicted that FGF18 was a predictive gene in the risk score model built by the authors to influence the survival difference of stage IV CRC patients (59). In terms of clinical survival prognosis of CRC, multivariate analysis of RNA-seq in 99 stage IV CRC patients predicted that FGF18 in CRC patients had significant changes in mRNA level and was significantly correlated with overall survival of CRC patients (60). Therefore, the clinical prognosis of CRC is closely related to the expression of FGF18.

In addition, obesity is established risk factors of CRC (61, 62), through DNA methylation spectrum(Illumina InfiniumHumanMethylation450 BeadChip) epigenetic changes, estimate the FGF18 was associated with a significant CRC, May be an important intermediate biomarker for obesity and CRC (63).

Effects of FGF18 on tissue and cell aspects of CRC. quantitative reverse-transcriptase polymerase chain reaction(qRT-PCR) analysis showed that FGF18 mRNA levels increased during the progression of CRC in 34 out of 38 CRC tissue samples. By IHC, FGF18 protein expression was increased in 20 CRC samples including mucosal, adenocarcinoma, primary, and metastatic cancers compared with normal non-cancerous mucosa. By endogeneously overexpressing FGF18 in human epithelioid colorectal adenocarcinoma cell lines Caco-2 and SW480, the number of cells was significantly increased. The cell cycle details of SW480 cells were analyzed by fluorescence activated cell sorter. The proportion of S-phase number of cells increased significantly with the stimulation of FGF18, and the angiogenic activity of SW480 cells was increased. Thus, FGF18 demonstrates that CRC cells can stimulate the proliferation and angiogenic activity of CRC cells by either paracrine or autocrine means (64).

By the construction of pUCFGF18TK vector plasmid and β-Gal enzyme linked immunosorbent assay(ELISA) assay, it was confirmed that FGF18 promoter was involved in driving reporter expression in CRC cell lines such as SW480 and HCT116 cells, compared with control group pUCTK without promoter, and cytotoxicity assay was performed. Apoptosis in both CRC cell lines was induced by the expression of the FGF18TK construct relative to the control construct (65). In conclusion, CRC progression was dependent on FGF18 expression at both cellular and tissue levels, *in vivo* and *in vitro*.

FGF18 is an important binding site in the oncogenic effects of CRC and is closely related to multiple pathways. For example, in the interaction of FGF18 with its receptor FGFR, in an *in vitro* irinotecan(IRI) -induced apoptosis model of CRC cells such as Caco2 and HCT116, FGF18 markedly enhanced cell viability as measured by SRB assay through the FGFR3-IIIc pathway and reversed IRI-induced cell cycle arrest as well as apoptosis in SW480 cells (66). By overexpressing FGFR3-IIIc in SW480 cells, FGF18 was found to increase the sensitivity of SW480 to migration and proliferation signals through the FGFR3-IIIc pathway upon stimulation with FGF18 (67).

In terms of cell surface receptors and classical signaling pathways. Western blot analysis showed that the phosphorylation of MAPK and PI3K/AKT in Caco-2 and LT97 cells was activated by human recombinant FGF18, which stimulated the proliferation and angiogenesis of CRC cells, and was also attenuated by the decreased intracellular expression of FGF18 (64). In terms of cell surface markers, the presence of CD44 markers on the surface of human colorectal adenoma cell line LT97 was classified. CD44 positive(CD44^(+)^) subpopulation had prolonged survival and growth ability in LT97 cells, and qRT-PCR analysis showed that FGF18 expression was increased in this subpopulation. Addition of artificial recombinant FGF18 protein increased the phosphorylation levels of ERK and GSK3β in LT97 cells and CD44-LT97 cells, and the phosphorylation level was higher in CD44-LT97 cells relative to LT97 cells.In addition, blocking Wnt pathway significantly decreased the mRNA and protein expression levels of FGF18 and affected the colony formation of CD44-LT97 cells (68). In conclusion, the CD44 marker on the surface of human colorectal adenoma cells as well as ERK can be mediated by FGF18 signaling, and Wnt can mediate FGF18 signaling activity (32, 64, 68–70). In terms of FGF18 gene transcription levels, beta-catenin (β-catenin) in association with TCF/LEF activates FGF18 gene transcription, and the FGF18 promoter contains a Tcf4 binding motif (32, 70).

Therefore, for effective prevention of colorectal cancer progression, selective inhibition of FGFR3-IIIc, WNT or MAPK and PI3K/AKT signaling pathways in CRC cells, as well as selective targeted therapy of CD44-positive subsets of CRC cells can be used. Further studies on more signaling pathways mediated by FGF18 and cell surface CD44 will provide new ideas for the diagnosis, treatment and prognosis of CRC.

### Hepatocellular carcinoma

Hepatocellular carcinoma (HCC) is the sixth most common cancer in the world, but its prevalence is increasing year by year (71). It is no less progressive and harmful than other cancers, yet there are few available treatment options (72, 73). Major risk factors for HCC include chronic viral hepatitis and alcoholic/non-alcoholic steatohepatitis, among others (74, 75). Advanced HCC is often diagnosed due to inadequate early screening, and the mortality rate is extremely high (75). Therefore, it is very important to explore the progression and mechanism of HCC to improve the prognosis of HCC.

FGF18 at the level of cell and tissue expression in HCC. In 2011, Christine’s team found by qRT-PCR that the mRNA level of FGF18 was significantly increased in HCC relative to normal liver tissue (76). Similarly, in 2017, Li’s team found that the mRNA and protein levels of FGF18 in tissue samples of HCC patients and human liver cancer cell lines HepG2 and HuH7 were significantly higher than those in normal liver tissue and human normal liver epithelial cell line LO2 (34). In addition, Christine’s team showed that serum starvation induced apoptosis in HepG2 or Hep3B human liver cancer cells, which was reversed by the addition of human recombinant FGF18 protein. These results suggest that FGF18 can improve the survival of HCC cells under hypoxia or nutrient deprivation. Moreover, knockdown of FGF18 in HepG2 and Hep3B cells resulted in a significant decrease in cell viability. It has also been found that FGF18 stimulates the proliferation of HCV-derived myofibroblasts (MF) through the production of vascular endothelial growth factor (vEGF), which in turn promotes the formation of new blood vessels in HCC (76). Taken together, FGF18 plays an important role in promoting migration, proliferation, invasion, apoptosis and angiogenesis in HCC tissues and cells.

In the process of exploring FGF18 and non-coding RNA mediated HCC progression. In 2007, while investigating the role of H19 non-coding RNA in HCC progression, Imad’s team found that enhancing H19 expression under hypoxic conditions significantly enhanced tumorigenic potential in Hep3B cell lines, whereas knockdown of H19 attenuated the tumorigenic potential. Interestingly, knockdown of H19 under hypoxic conditions similarly reduced FGF18 by semi-quantitative RT-PCR analysis, indicating that FGF18 is a downstream gene when H19 non-coding RNA promotes HCC progression under hypoxic conditions (77). Moreover, in addition to finding that FGF18 is highly expressed in HCC tissues and cell lines, Li’s team also found that FGF18 mediates the regulation of miR-139 on HCC progression. These results indicated that FGF18 could mediate H19 non-coding RNA and miR-139 to promote the tumorigenic potential of HCC (34).

In terms of cell surface receptors and classical signaling pathways. In 2018, Guo’s team analyzed mRNA related data in the GSE 1898 database when studying the progression of ribosomal protein s15a(RPS15A) (78) in HCC, and found that FGF18 was significantly correlated in it. RPS15A can increase the expression of FGF18 in HCC cells through Wnt/β-catenin signaling pathway. Further, by co-culturing HuH7 with human umbilical vein endothelial cells(HUVEC), it was found that FGF18 promoted HCC angiogenesis by enhancing the FGFR3 pathway as well as the phosphorylation level of AKT/ERK proteins, and was regulated upstream by Wnt/β-catenin signaling (69, 70, 79). In summary, FGF18 can accelerate the progression of HCC in a variety of ways, and these targets can provide us with new ideas for improving the prognosis of HCC.

### Ovarian cancer

Ovarian cancer (OC) is the fifth leading cause of high cancer mortality among women in the United States (80), of which 90% are epithelial cancers. Although the incidence and mortality of OC are decreasing year by year (81), inadequate early screening often results in advanced cancer at the time of diagnosis, making treatment a major challenge. Moreover, the tumor heterogeneity of different subtypes of OC is very high, which is also the reason that the treatment work is very difficult, etc., so the prognosis is often very poor (82–84). In summary, it is still very important to find new therapeutic targets, optimize treatment regimens, and improve prognosis.

FGF18 expression in OC tissues and cells. A search for human EST computer expression analysis derived from FGF18 mRNA using the BLAST program predicted FGF18 mRNA expression in GC (11). From including Zhang database, SPD (secreted protein database), GPRCDB (A Molecular-Specific Information System for G Protein-Coupled Receptors), AmiGO (Gene Ontology database), Uniprot secreted proteins and Signal Peptide Website (An Information Platform for Signal Sequences and signals Peptide), eight public databases and Affymetrix U133 Plus 2.0 probe identifiers were used to establish the secretome, and then transcriptome analysis showed that FGF18 expression was significantly increased in OC tissues. A significant increase in serum FGF18 expression in OC patients relative to matched normal controls was verified by ELISA (85). In addition, transcriptome sequencing analysis of a benign ovarian epithelial tumor cell line MCV152 and an ovarian serous cancer cell line SKOV-3 identified FGF18 as an up-regulated gene, which was validated in WB (86). FGF18 was predicted to discriminate benign or malignant epithelial ovarian cancer (EOC) at the transcriptional level by differential gene expression analysis of epithelial ovarian cancer (EOC) from the comprehensive gene expression database, Cancer Science Institute of Singapore OC Database (CSIOVDB) (87).

In terms of clinical relevance of FGF18, FGF18 is a valid diagnostic marker in OC. In 103 OC patient samples, FGF18 expression was increased in serous and mucinous ovarian cancers by IHC as well as analysis of clinicopathological variables and patient outcomes, and was similarly significantly increased from adenoma to borderline tumor to type I cancer and then from type I to type II cancer (88). In 2013, Wei’s team predicted that overexpressed FGF18 was an independent marker of poor prognosis in OC using high-throughput genomic technology. In OC tissues and SKOV3 et al. ovarian cancer cells, FGF18 mRNA and protein levels were increased in serous ovarian tumors relative to normal ovarian epithelium as analyzed by qRT-PCR and immunohistochemistry (IHC) (33). Therefore, it can be determined that FGF18 is up-regulated in OC tissues or cells and has a significant correlation with the prognosis and progression of OC. Tagging single-nucleotide polymorphisms (SNPS) (89) were extracted from genes encoding FGF or FGFR, the association of each SNP with OC was determined in genetic models (dominant, recessive, and additive), where SNP rs3806929 of FGF18 was significantly associated with treatment response after platinum-based chemotherapy (90).

To explore the correlation between the prognosis of OC after the expression of FGF18 and tumor angiogenesis as well as the infiltration and polarization of tumor-associated macrophages. Tumor microvessel density (MVD) increased with FGF18 expression in serous ovarian cancer, and FGF18 and MVD increased significantly in type I to type II cancer progression (88). In addition, Wei’s team further found that FGF18 could enhance the invasion of OC cells and regulate the tumor microenvironment *in vitro* and *in vivo* experiments on OC designed SKOV3 cell line and SCID mouse xenograft, especially enhance the angiogenesis of OC and enhance the infiltration and M2 polarization of tumor-associated macrophages. In turn, it promotes the progression of OC (33). FGF18 is therefore identified as an important therapeutic target in the control of OC progression.

In exploring the progression of the tumor-promoting mechanism of FGF18 in epithelial ovarian cancer. gene set enrichment analysis (GSEA) prediction and validation of SKOV3, KURAMOCHI, OVTOKO, and HEY et al. ovarian cancer cells showed that FGF18 could function through the PAX8-FGF18 axis pathway. It promotes OC cell migration in an autocrine manner (91). In addition, FGF18 can also regulate the migration, invasion and tumorigenicity of OC cells A224 through the NF-κB pathway, and increase the cytokines IL-1A, IL-6, IL-8 and chemokines CXCL1 and CXCL2 (33). Aldehyde dehydrogenase 1 (ALDH1) expression in human ovarian cancer cell line SKOV3 was divided into ALDH1 (high) and ALDH1 (low) subgroups (92–95). ALDH1 (high) cells exhibit cancer stem cell (CSC) properties such as phenotypic diversity and *in vivo* tumorigenicity (96–99), and by ovarian CSC marker enrichment analysis and qPCR analysis, FGF18-FGFR3 expression was increased in the ALDH (high) subgroup (100). Therefore, FGF18 has a significant relevance in OC cancer stem cell properties, and the pathways through which FGF18 mediates OC progression are very rich, and there are many more sites that still need to be explored.

### Breast cancer

Breast cancer is by far the most common cancer in the world, accounting for about 30% of all cancers in women (101, 102). Risk factors include age, smoking, diet, pregnancy history, family history, history of breast cancer, hormone therapy, and genetic mutations (103–107). Surgery, radiotherapy, and chemotherapy are still the most basic options for breast cancer treatment (108, 109). However, neoadjuvant combination therapy with targeted agents, such as CDK6 and CDK4 inhibitors, has been gradually integrated into the on-track treatment regimen, reflecting the complexity of breast cancer treatment today (78, 101, 110, 111). Therefore, it is still difficult to explore new target sites and treatment options and improve the prognosis of breast cancer patients.

In terms of clinical relevance of FGF18, FGF18 is a valid diagnostic marker in breast cancer. Obesity is one of the major risk factors for breast cancer (101, 112, 113). In DNA methylation spectrum epigenetic changes, predict FGF18 significantly associated with breast cancer, breast cancer is likely to be obese and important intermediate of biomarkers (63). Breast cancer-related genes were screened in the GEO database and found that FGF18 was statistically significant with the progression of breast cancer. By Kaplan Meier analysis, defining the risk of breast cancer recurrence, FGF18 was significantly correlated with disease-free survival (DFS) of breast cancer.

FGF18 expression in OC tissues and cells. In qRT-PCR, an evaluation based on FGF18 mRNA levels in 261 invasive breast cancer patients validated the previous prediction (114). Analysis of FGF18 gene and protein levels by Oncomine cancer Genome Atlas database and Human Protein Atlas database showed that FGF18 expression was increased in breast cancer compared with normal tissues. By IHC staining, FGF18 protein levels were significantly increased in breast cancer tissues. Interestingly, the mRNA levels of MCF-7, T47D, SK-BR-3, and MDA-MB-453 human breast cancer cell lines were significantly increased under hypoxic conditions by qPCR (115).

In terms of exploring the progress of FGF18 in breast cancer cells. Stimulation of human breast cancer cell line MDA-MB-231 with FGF18 recombinant protein enhanced its proliferation ability and increased the number of colonies formed. Regarding the cell cycle, the percentage of G0/G1 phase was increased and the percentage of S phase was decreased in MDA-MB-231 cells relative to the control group without FGF18 recombinant protein. The proliferation, migration and invasion of human breast cancer cell line MDA-MB-231 with FGF18 knockdown or overexpression were significantly affected by WB, qPCR and other technologies (35).

In exploring the mechanism of FGF18 in breast cancer progression. In the same human breast cancer cell lines MDA-MB-453 and SK-BR-3, the mRNA levels of some proliferation-related genes, such as CCND2, CDK2 and Ki67, were significantly up-regulated when stimulated by recombinant FGF18 protein. In addition, the mRNA levels of tumor migration factors TGF-β, MMP-9 and MMP-2 as well as epithelial-mesenchymal transition (EMT) markers N-cadherin, vimentin, Snail-1, Snail-2 and TIMP-1 were also significantly up-regulated by FGF18 stimulation. Western blotting showed that FGF18 treatment enhanced the phosphorylation of Akt-Ser473, Akt-Thr308 and GSK3β-Ser9 in MDA-MB-453 and SK-BR-3 cells. Therefore, FGF18 regulates breast cancer cell growth and tissue progression through Akt-GSK3β-mediated β-catenin signaling. In addition, by chromatin immunoprecipitation assay (ChIP) and quantitative ChIP assay analysis, β-catenin was associated with the promoter of proliferation-related genes (CCND2, CDK2, and Ki67) as well as migration-related genes (TGF-β, MMP-9, and MMP-2) and EMT markers (N-cadherin, vimentin, Snel-1, and Vimentin). Snail-2 and TIMP1) can be bound in MDA-MB-453 cells (115). By WB, qPCR and other techniques, we found that FGF18 could enhance the proliferation, migration and invasion of human breast cancer cells MDA-MB-231 through ERK phosphorylation and c-Myc signaling pathway activation (35).

In the *in vivo* experiment, subcutaneous injection of FGF18 knockdown or overexpression MDA-MB-231 cells into nude mice showed that the tumor size of FGF18 overexpression xenograft model was significantly larger than that of the control or FGF18 knockdown group (35). In summary, FGF18 helps us to have a more systematic understanding of the genetic and molecular protein aspects in the prediction and treatment of breast cancer progression.

### Lung cancer

Lung cancer is currently the second most common cancer in the world, and also the cancer with the highest mortality, and its incidence is steadily increasing year by year (116). Among them, the most important risk factor is smoking, and the incidence gradually increases with age, and the prognosis gradually deteriorates (117–119). Currently, the main treatment methods include surgery, radiotherapy, chemotherapy, and immunotherapy (120–122). Therefore, the adjustment of early lung cancer screening procedures and methods (123), the implementation of practices to reduce the risk of lung cancer, and the exploration of treatment processes to improve the prognosis of lung cancer are our top priorities.

Regarding the prediction of differential expression and clinical relevance of FGF18 in lung cancer and normal lung tissues. The BLAST program searches for human EST computer expression analysis derived from FGF18 mRNA and predicts that FGF18 mRNA is expressed in lung carcinoids (11). In 2022, Guo et al. identified FGF18 as a differentially expressed gene that could be up-regulated by overexpression of Histone deacetylases(HDACs) (124, 125) in human poorly differentiated lung adenocarcinoma SK-LU-1 cells by RNA-seq transcriptome analysis. Western blot was used to verify that the protein expression level of FGF18 was increased in lung cancer cells due to the overexpression of HDACs. By Kaplan-Meier analysis of 319 patients, FGF18 was associated with poor prognosis in NSCLC patients. The expression level of FGF18 protein in tumor tissues of 12 non-small cell lung cancer (NSCLC) patients was significantly higher than that in adjacent non-tumor tissues, and with the increase of FGF18 expression, the prognosis, tumor differentiation and tumor-lymph node metastasis (TNM) stage of NSCLC patients were all in the poor direction (126).

In terms of FGF18 on the progression of lung cancer cells and tissues. Knockdown or overexpression of FGF18 in human non-small cell lung cancer cell A549 significantly reversed the effects of HDACs on the proliferation, migration and cell cycle of lung cancer cells. In the xenograft model, FGF18 overexpression significantly increased the weight of subcutaneous tumors in nude mice, while FGF18 knockdown had the opposite effect. Injection of FGF18 overexpression cells significantly increased the incidence of lung metastasis and the number of pulmonary metastatic nodules in nude mice, as measured by tail vein metastasis, with stronger fluorescence imaging signals (126). In addition, stimulation of human lung cancer cell line H460 with recombinant FGF18 protein significantly promoted the proliferation of H460 cells. In terms of cell cycle, FGF18 increased the proportion of cells in G0/G1 phase and decreased the proportion of cells in S phase or G2/M phase. In A wound healing assay, the migration ability of H460 cells was significantly enhanced after stimulation with recombinant FGF18 protein. Western blotting and qPCR confirmed that FGF18 increased the mRNA and protein expression levels of MMP26 (127).

In terms of the mechanism and pathway of FGF18 on lung cancer progression. As verified by WB technology, FGF18 promoted the proliferation and migration of H460 cells by enhancing the phosphorylation levels of ERK and p38 signaling pathways (127). FGF18 is known to be inextricably related to β-catenin (10, 69, 70). Through IF, co-IP, and nucleocytoplasmic protein separation assays, it was proved that HDAC7 promoted the proliferation and migration of NSCLC by reducing the phosphorylation of β-catenin at Ser45 and the acetylation of β-catenin at Lys49 and promoting its nuclear translocation and accumulation, then combined with TCF4 to regulate FGF18 to promote the proliferation and migration of NSCLC (126).

The interaction between FGF18 and FGFR in lung cancer progression was investigated. Fibroblast growth factor receptor 1 (FGFR1) Fc fusion protein (FP-1039) (128) inhibited the growth of five lung cancer cell lines NCI-H1581, NCI-H520, DMS114, NCI-H1703 and DMS53. It also suppressed tumor formation in subcutaneous tumor models of five lung cancer cell lines. Interestingly, the expression of FGF18 at mRNA level detected in tumors of xenograft models by qRT-PCR was positively correlated with the antitumor effect of FP-1039, and surface plasmon resonance (SPR) spectroscopy showed that FP-1039 could be bound to FGF18 with high affinity (129). suggesting that FGF18 may affect lung cancer progression through the FGFR1 pathway. In conclusion, FGF18 plays an important role in promoting the growth and tissue progression of lung cancer cells through FGFR1-ERK/p38 pathway. FGF18 and β-catenin are closely related to regulate the occurrence and development of NSCLC.

### Endometrial cancer

Endometrial cancer (EC) is the most common gynecological malignancy and the fifth most common malignancy in women worldwide (5, 130, 131). Major risk factors for EC include obesity, age, diabetes, early menarche, and estrogen exposure (132–136). Ultrasonography and endometrial biopsy are the first choice for diagnosis (137). Treatment options should be judged according to the development of the disease, including surgery, lymph node dissection, and adjuvant radiotherapy and chemotherapy (138–141). Its pathogenesis and treatment options are complex. Therefore, it is very important to further study its mechanism to improve its diagnosis and prognosis.

In terms of exploring the clinical relevance of FGF18 to EC. Analysis of differential gene expression between normal endometrial tissue and EC tissue by microarray showed that FGF18 was upregulated in EC tissue, which was then verified by RT-PCR that the mRNA level of FGF18 was increased by 10.8-fold in EC (36, 142). Furthermore, By Agilent Whole Human Genome Array GeneChip microarray analysis, FGF18 was significantly upregulated in ESC cultures in response to estrogen stimulation. FGF18 expression is higher in primary endometrial stromal cells (escs) of endometrioid EC (EEC, type I EC) than in normal endometrium (NE), escs of type II EC and endometrial atypical hyperplasia (EAH) (143).

In exploring the progression of FGF18 in EC cells. By Agilent Whole Human Genome Array GeneChip microarray analysis, FGF18 was significantly upregulated in ESC cultures in response to estrogen stimulation. With accelerated proliferation of endometrial epithelial cells, FGF18 mRNA and protein expression levels increase (144). After knockdown of FGF18, the proliferation and invasion of EC cell lines ishikawa and Hec-1A were significantly reduced (143). In summary, FGF18 promotes EC cell progression.

In terms of exploring the mechanism of FGF18 progression with FGFR as well as the classical pathway in EC. FGF18 can first bind to FGFR2 and FGFR3, and then promote the viability of ishikawa cells by enhancing the phosphorylation levels of AKT and ERK downstream, and the activation of Survivin and CD44V6 expression is closely related to the FGFR/FGFR3 pathway of FGF18 (143). In addition, FGF18 expression levels can be inhibited by the tissue selective estrogen receptor modulator bazedoxifene (BZA), which in turn decreases the proliferative capacity of EC (36). In summary, FGF18 may have multiple pathways to promote EC progression at transcriptional and protein levels.

### Renal carcinoma

The prevalence of renal cell carcinoma accounts for 2% of all cancers in the world, but its incidence and mortality are increasing year by year. Clear cell renal cell carcinoma (ccRCC) accounts for 75% of all renal cancers and is the most common subtype of renal cancer (145, 146). In particular, the prognosis of metastatic ccRCC is extremely poor, and the survival rate of patients with more than 5 years of disease age is less than 10%. So far, no better treatment options have been found except surgery (147, 148). Therefore, it is very important to explore the mechanism of renal cell carcinoma to find new methods to improve the treatment and prognosis.

In terms of the expression of FGF18 in ccRCC, TCGA database analysis showed that FGF18 was predicted to be down-regulated in ccRCC tissues, and the high expression of FGF18 was associated with a good prognosis of ccRCC. Protein and mRNA levels of 769-P and A498 as well as human renal carcinoma tissues were validated (149).

In terms of the mechanism of ccRCC progression. Overexpression of FGF18 in 786-O and 769-P cells significantly attenuated the proliferation and invasion of ccRCC cells and xenograft models in mice. Moreover, it can inhibit ccRCC cell proliferation and epithelial-mesenchymal transition by reducing the phosphorylation level of PI3K/AKT and the protein expression of EMT-related markers and transcription factors.Moreover, the protein levels of E-cadherin were increased, while the protein levels of Vimentin and N-cadherin were decreased by overexpression of FGF18, which was verified in the xenograft model *in vivo* (149). Thus, FGF18 can inhibit the progression of ccRCC.

### Synovial sarcoma

Synovial sarcoma (SS) is a soft tissue sarcoma (STS) (150) that accounts for 5% of all STS and is the most common sarcoma subtype (151, 152). In contrast to other STS, SS is very sensitive to chemotherapy, and anthracyclines and ifosfamide are the conventional first-line chemotherapy for SS (153). Its pathogenesis and treatment are complex, and the prognosis becomes worse with increasing age (154, 155). Therefore, it is important to further investigate its mechanism to improve its diagnosis and prognosis.

In terms of FGF18 progression on SS cells as well as tissues. By RT-PCR, FGF18 gene was detected in SS cell lines and SS tumor tissues, and the expression level was significantly higher than that in other types of soft tissue sarcomas such as pleomorphic liposarcoma (PLS) and leiomyosarcoma (LMS). The expression of FGF18 protein was detected in the culture supernatant of SS cell lines as verified by WB, although the protein level was not completely consistent with the RNA expression level (156).

In terms of exploring the role and mechanism of FGF18 in SS. Stimulation of human synovial sarcoma cell line HS-SY-II with recombinant FGF18 protein increased the dependence of the cells on FGF18 and enhanced the phosphorylation levels of ERK, P38 and FGFR3 proteins in human synovial sarcoma cell line HS-SY-II. In exploring growth inhibitory factors in SS, ERK phosphorylation was completely inhibited after inhibition of FGFR autophosphorylation using SU5402 (157, 158), whereas p38 phosphorylation was not affected. Regarding the cell cycle, SU5402 induced G1 arrest. In addition, injection of PD166866 (159, 160) significantly inhibited ERKphosphorylation and tumor growth in a mouse model of SYO-1. Thus, exogenous FGF18 promotes synovial sarcoma growth through FGFR3 and ERK pathways in synovial sarcoma cell lines (156).

### Other cancers

In the treatment of cervical cancer. By gene analysis of 14 cervical cancer cell lines selected from the GDSC (Genomics of Cancer Drug Sensitivity) database, FGF18 significantly reduced the resistance of patients to cisplatin (161, 162). FGF18 may play an important role in guiding platinum-based therapy of cervical cancer.

In the progression of testicular tumors. Microarray analysis of MA-10 mouse Leydig tumor cells (163, 164) showed that cordycepin(3′-deoxyadenosine) (165, 166) downregulated FGF18 mRNA levels in testicular Leydig tumor cells. In addition, the IPA-MAP pathway prediction analysis showed that cordycepin inhibited the proliferation of MA-10 cells by inhibiting the FGFs/FGFRs pathway (167). In conclusion, FGF18 plays an important role in promoting the proliferation of Leydig tumor cells.

In the progression of bladder cancer. In genome-wide expression analysis of bladder cancer cells treated with demethylating agents 5-aza-2’-cytidine and zebularine (168–170), FGF18 expression was downregulated in eight bladder cancer cell lines. Thus, FGF18 expression status in bladder cancer cell lines is downregulated by methylation of CpG residues located near the gene promoter region, with consequent impact on bladder cancer progression (171).

In the diagnosis of pheochromocytoma (PCC) and paraganglioma (PGL) (172–174). By TCGA database analysis, actin cytoskeleton regulated genes were significantly upregulated in PCC/PGL with metastasis compared with PCC and PGL without metastasis, including FGF18. Thus, FGF18 may play an important role in the progression of tumor metastasis in PCC or PGL (175).

In the progression of malignant pleural mesothelioma (MPM). By genome-wide gene expression microarray analysis, 12 MPM cell lines showed increased expression of FGF18 compared with non-malignant mesothelial cells. FGF18, FGFR1 and FGFR2 mRNA and protein levels were significantly increased, as validated by qPCR and IF. FGF18 and FGFR1 protein levels were increased. Interestingly, the clonogenicity, proliferation, spheroid growth and migration ability of MPM cells were significantly decreased by FGFR1 inhibition. Moreover, FGFR1 inhibition induced MPM cell apoptosis through PI3K/AKT and MAPK/ERK pathways and inhibited MPM progression *in vivo*. Inhibition of FGFR1 enhances the sensitivity of MPM to cisplatin and radiation (176–178). However, whether FGFR1 is a direct target of FGF18 in MPM remains to be explored.

In the progression of melanoma (179, 180). Expression of FGF18 and four FGFR genes was detected in 12 normal melanoma cell lines (NM) and melanoma cell lines by RT-PCR. Interestingly, the expression of FGF18 in different melanoma cell lines was different. The expression of FGF18 in VM7, VM10, VM21, VM24 and other melanoma cell lines was less than that in NM, but the expression of FGF18 in VM23 and VM31 was more than that in NM. FGF18 protein expression was significantly lower in VM21 and VM24 than in NM (181). Therefore, the role of FGF18 and FGFR in melanoma progression remains to be explored.

In the progression of human teratomas (182). Survival and cell viability of the human embryonal carcinoma derived cell line Tera 2 were increased by FGF18 stimulation *in vitro (* 183). In addition, in juvenile nasopharyngeal angiofibroma (JNA) (184, 185), FGF18 expression in endothelial cells of JNA was positively correlated with its expression in stromal cells (186), as verified by qPCR by gene expression analysis. In pediatric ependymoma studies, FGF18 was predicted to be an ependymoma-associated gene and associated with cell cycle by methylation and expression microarray data analysis (187). However, how FGF18 specifically directs tumor progression in embryonic and infantile tumors remains to be explored.

## Molecular structure and homology of FGF18

The human FGF18 gene encodes a protein sequence consisting of 207 amino acids with a molecular mass of approximately 23kDa (188, 189). FGF18 homologues are members of the FGF family with N-terminal signal peptides. The homologous region of FGF18 in the human genome is located at FBXW11 (βTRCP2 or BTRC2) -FGF18-NPM1 on human chromosome 5q35.1. The total amino acids of human and rat FGF18 were also confirmed to be identical (11).

In addition, FGF18 promoter has double TCF/LEF binding sites, and it is worth noting that, FGF18 promoter can bind to the complex formed by Runx2 and Lef1/TCF4 to form a complex binding site, which in turn enhances FGF18 expression by normalizing Wnt signaling (11, 190). Thus, FGF18 orthologs are evolutionarily conserved targets of the canonical Wnt/β-catenin signaling pathway based on comparative genomics of the 5’ -promoter region of FGF family genes, FGF18 and WNT signaling pathways are networked together during carcinogenesis and embryogenesis (10, 11, 69, 70, 191).

## Interaction of FGF18 with its receptor

### Importance of FGFR for tumor progression

FGFR is a group of receptors that span the cell surface membrane by a single channel encoded by a special independent gene. This receptor is a polypeptide structure, which is linked together by dehydration and condensation of several amino acid molecules. So far, five members of the FGFR family have been found (FGFR1-5), and their genes have homology in terms of coding sequence, which also lays the foundation for the similarity of their functions and expression (192). With the in-depth study of the relationship between the structure, function and disease of FGFR, it can provide new treatment methods for a variety of neoplastic diseases and make it possible to conquer cancer.

### Structural basis for the interaction of FGF18 with FGFR

Regarding the mechanism of action of FGF18 with FGFR. Fgfr1-4 has a similar structure with three basic domains: extracellular, membrane and intracellular. The ligand binding sites containing three immunoglobulin-like structures (IgI,IgII, and IgIII) are located outside the cell, the one-way transmembrane structure is located on the cell membrane, and the tyrosine kinase structure is located inside the cell.The extracellular domain can bind to heparin or heparin sulfate (HS) proteoglycans, and it has been shown that this tight binding not only enhances FGF-FGFR complex binding, but also interacts with neighboring FGF-FGFR complexes to promote FGFR dimerization (193).

The binding relationship between IgIand ligand was not tight. There is a positive correlation between the exonic region of IgII,IgIII and Igii-Igiii linker and the ligand-binding reaction (194). There is an acidic amino acid base sequence between the IgI-IgII linker, which is called the “acid box”. The loss of both will not affect the binding function of antibody (FGFR) and ligand (FGF) (195), and even some studies have shown that the loss of IgI and acid box can improve the binding affinity of receptor and ligand (196). Alternative gene splicing of Ig III also gives rise to different receptor subtypes, named FGFRIIIb and FGFRIIIc (197). For example, FGF18 can significantly enhance CRC cell viability and reverse cell cycle arrest and apoptosis induced by IRI through FGFR3-IIIc pathway (66).

### Signaling activation and signaling transduction of FGF18 interacting with FGFR

FGF binds specifically to FGFR outside the cell membrane, inducing receptor dimerization, and then one of the dimers phosphorylates the other, which is called autophosphorylation of the receptor. This reaction fully activates the tyrosine kinase domain of the receptor intracellular domain, and the activated tyrosine kinase initiates a cascade activation reaction. “Multiple tyrosine residues within the fibroblast growth factor receptor are activated in a multistage manner, resulting in an increase in tyrosine kinase activity of as much as 500 - to 1,000-fold, which is the basis for continued down-transduction of the signal (198).”

The activated FGFR can then direct the signal along different intracellular transduction pathways. For example, with the activation of FGF18-FGFR2-c-Jun-YAP1 axis, the clinical prognosis of GC moves in an unfavorable direction (53); FGF18 promotes the proliferation of lung cancer cell lines and tumor formation in subcutaneous xenograft models through FGFR1-ERK pathway (127, 129); FGF18 promotes angiogenesis and tumor growth in HCC by enhancing FGFR3 pathway and phosphorylation of AKT-ERK protein, and is regulated upstream by Wnt/β-catenin signaling pathway (79).

## Conclusion and perspectives

### FGF18 expression and prognostic correlation in cancer

**Figure 1:**
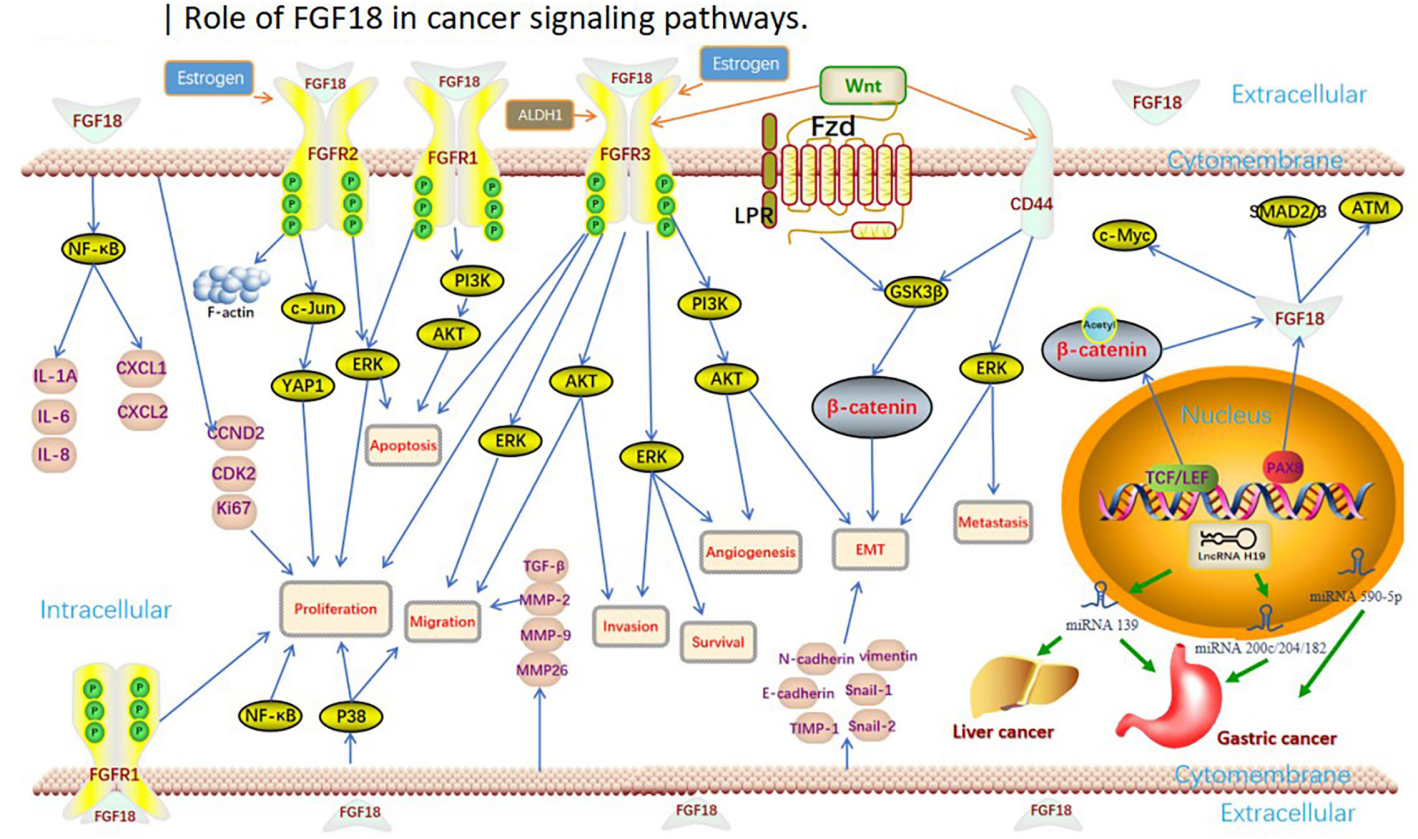
Role of FGF18 in cancer clinical significance and functions.

FGF18 has been shown to be not only limited to regulating the metabolism and development of human normal tissues and organs, but also an important oncogene, which plays an important role in guiding the progression of cancer in digestive, reproductive, respiratory and urinary organs or systems (31), and has a significant impact on the diagnosis and prognosis of cancer (**Figure 1**).

In the cancers that have been discovered so far, it can be seen that the expression of FGF18 is elevated in the tissues or cells of most cancers (**Table 1**). With the expression and stimulation of endogenous or exogenous FGF18, it can significantly promote the development of malignant phenotypes such as proliferation, migration and invasion of most tumors (**Table 2**). However, this is not the case for all cancers. For example, FGF18 is predicted to be down-regulated in ccRCC tissues through TCGA database analysis, and high expression of FGF18 is associated with a good prognosis of ccRCC (149). However, FGF18, as an important factor promoting human tissue and cell metabolism, can inhibit the progression of tumor cells in renal cancer. It is possible that FGF18 has special binding factors and targets in ccRCC cells, or unknown secreted proteins interact with FGF18 in tissues., thus, the expression of FGF18 in ccRCC and tumor progression were inhibited jointly.

**Table 1:** Summary of the mechanism studies of FGF18 in tumors.

Tumor	Expression	Mechanism	Ref
Gastric Cancer	up	LncRNA H19-miRNA 200c/miRNA 139, lncRNA H19-miRNA 204/miRNA 182, FGF18-FGFR2-c-Jun-YAP1, SMAD2/3, ATM, F-actin, ERK-MAPK	(43, 44, 46, 47, 53)
Colorectal Cancer	up	CD44, ERK-MAPK, PI3K -AKT, GSK3β, Wnt/β-catenin-FGFR3-IIIc	(11, 32, 59, 60, 64–68, 70)
Hepatocellular Carcinoma	up	LncRNA H19- miRNA 139, Wnt/β-catenin-FGFR3, AKT, ERK/MAPK	(34, 76, 77, 79)
Ovarian Cancer	up	ALDH1, PAX8-FGF18, NF-κB-IL-1A/IL-6/IL-8, NF-κB-CXCL1/CXCL2	(33, 85–88, 91, 100)
Breast Cancer	up	CCND2, CDK2, Ki67, TGF-β, MMP-9, MMP-2, N-cadherin, vimentin, Snail-1, Snail-2, TIMP-1, ERK/MAPK, c-Myc, Akt-Ser473, Akt-Thr308, Wnt/β-catenin, GSK3β-Ser9	(35, 63, 114, 115)
Lung Cancer	up	HDAC7-Wnt/β-catenin(acetylation level at Lys49 and decreased phosphorylation level at Ser45, FGFR1-ERK, P38, MMP26,	(126, 127, 129)
Endometrial Cancer	up	Estrogen, FGFR2/FGFR3, AKT, ERK, Survivin, CD44V6	(36, 142, 143)
Renal Carcinoma	down	PI3K/AKT, E-cadherin, Vimentin, N-cadherin	(149)
Cervical Cancer	not mentioned	Not mentioned	(162)
Bladder Cancer	not mentioned	Methylation of CpG	(171)
Synovial Sarcoma	up	FGFR3, ERK, P38	(156)
Pheochromocytoma/Paraganglioma	up	Not mentioned	(175)
Melanoma	up/down	Not mentioned	(181)
Teratomas	up	Not mentioned	(183)
JNA	up/down	Not mentioned	(186)
Pediatric ependymoma	up	Not mentioned	(187)
Testicular Leydig Tumor	up	FGFRs	(167)

**Table 2:** FGF18 with tumor type,phenotype or significance and functions in tumors.

Tumor	Tumor tpye	Phenotype or significance	Function	Ref
Gastric Cancer	carcinomas	Overall survival, Tumorigenesis, Tumor size, Cell cycle, Advanced stages	Proliferation, apoptosis	(43–47, 53)
Colorectal Cancer	carcinomas	Overall survival(stage IV), Cell cycle, Activity of angiogenesis, Cell viability, Colony formation	Proliferation, apoptosis, migration, survival, growth	(59, 60, 64–68)
Hepatocellular Carcinoma	carcinomas	Tumor size, Cell viability, Colony formation, Tumor angiogenesis, Propagation of myofibroblasts	Proliferation, apoptosis, migration, invasion, angiogenesis	(34, 76, 77, 79)
Ovarian Cancer	carcinomas	Overall survival, Tumorigenesis, Tumor angiogenesis, Tumor atypia, Tumor microvessel density, Infiltration and M2 polarization of macrophages, Sensitivity to cisplatin chemotherapy, Characteristics of cancer stem cells	Invasion, migration	(33, 87, 88, 90, 91, 199)
Breast Cancer	carcinomas	Overall survival, Disease-free survival, Colony formation, Cell cycle, Epithelial-mesenchymal transition	Proliferation, invasion, migration, growth, metastasis	(35, 114, 115)
Lung Cancer	carcinomas	Tumor differentiation, TNM stage, Cell cycle, Tumor size, Incidence of pulmonary metastasis and number of pulmonary metastatic nodules, nuclear transfer	Proliferation, migration, distant metastasis	(126, 127, 129)
Endometrial Cancer	carcinomas	Estrogen dependence, Cell viability	Proliferation, invasion,	(36, 143, 144)
Renal Carcinoma	carcinomas	Overall survival, Tumor size, Tumor weight, Tumor grades, EMT, Lung metastasis, Colony formation, Immunoreactivity	Proliferation, invasion, distant metastasis	(149)
Cervical Cancer	carcinomas	Sensitivity to cisplatin chemotherapy	Not mentioned	(162)
Bladder Cancer	carcinomas	Not mentioned	Not mentioned	(171)
Malignant pleural mesothelioma	carcinomas	Not directly mentioned	Not mentioned	(178)
Synovial Sarcoma	sarcoma	Tumorigenesis, Tumor size, Cell cycle, FGF18 dependence	Proliferation	(156)
Pheochromocytoma/Paraganglioma	tumors	Malignant tumor metastasis	Metastasis	(175)
Melanoma	tumors	Different expression among different tumor cells	Not mentioned	(181)
Teratomas	tumors	Cell survival rate, Cell viability	Survival	(183)
Juvenile nasopharyngeal angiofibroma	tumors	Differential expression in endothelial versus stromal cells in JNA	Not mentioned	(186)
Pediatric ependymoma	tumors	Cell cycle	Not mentioned	(187)
Testicular Leydig Tumor	tumors	Cordycepin	Proliferation	(167)

In addition, the effect of FGF18 is also reflected in the correlation with radiotherapy, chemotherapy and other drugs on cancer prognosis (**Table 2**). For example, FGF18 expression was significantly elevated in MPM, and FGFR 1 inhibition inhibited MPM progression and increased MPM sensitivity to cisplatin and radiation (176–178); FGF18 significantly reduced the resistance of cervical cancer patients to cisplatin (162). In a geneticmodel, the FGF18 SNP rs3806929 was significantly associated with treatment response to OC after platinum-based chemotherapy (90). After years of development, the application of cytotoxic drugs has developed rapidly. With the use of drugs, overcoming drug resistance has become an difficult problem to overcome, and FGF18 may be a potential target to solve the sensitivity and resistance of tumor chemotherapy drugs.

### Regulatory mechanisms of FGF18 in cancer

**Figure 2:**
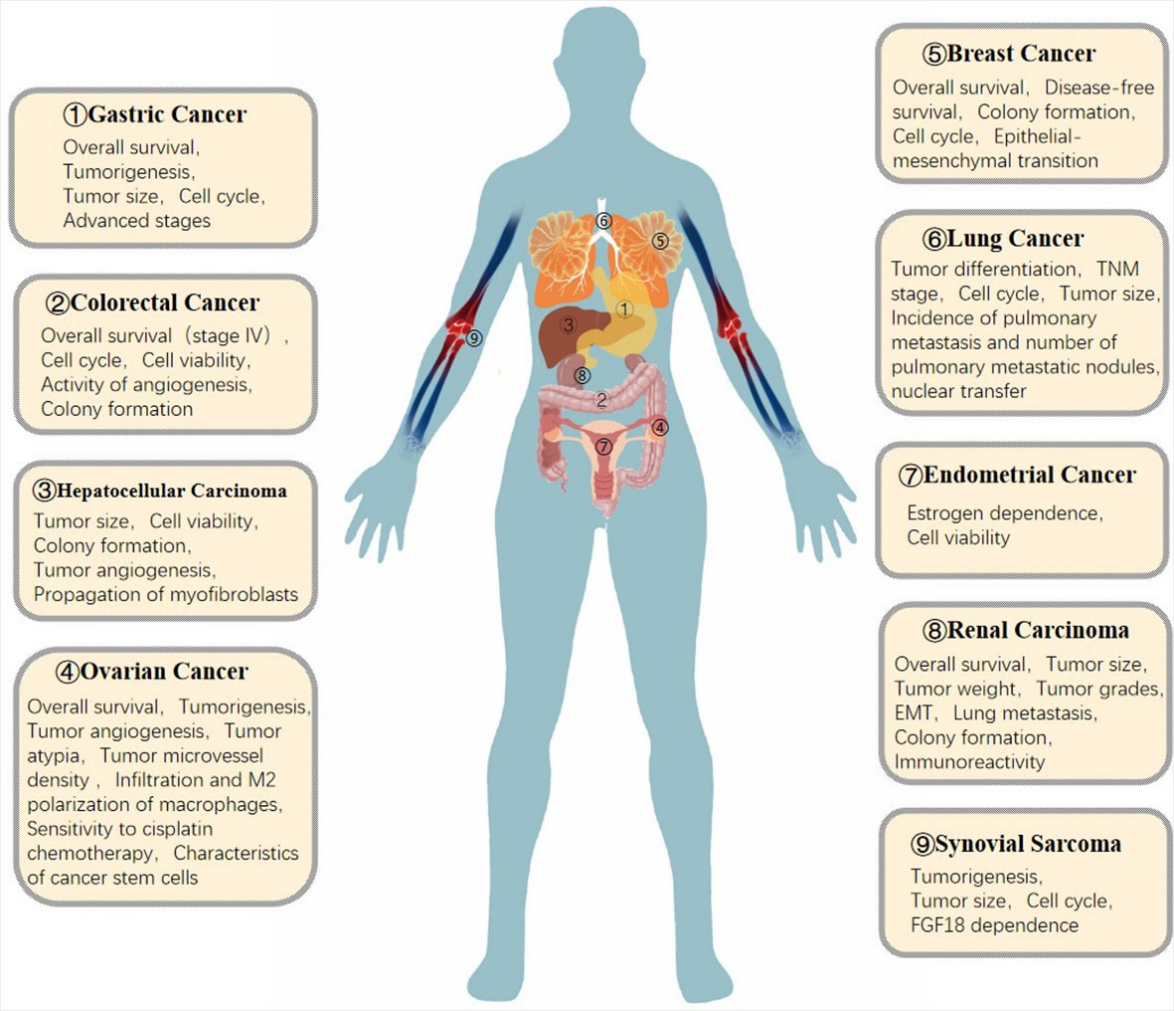
Role of FGF18 in cancer signaling pathways.

The regulatory mechanism of FGF18 is very complex, and FGF18 has different regulatory pathways and modes in different tumors (**Figure 2**). For example, FGF18 promotes GC cell growth through autocrine activation of SMAD2/3 and inhibition of ATM signaling (43), and with the activation of FGF18-FGFR2-c-Jun-YAP1 axis, the clinical prognosis of GC is adverse (53). In breast cancer, FGF18 can activate the expression of tumor migration-related genes such as TGFβ and MMP-2, proliferation-related genes such as CCND2 and CDK2, and EMT markers such as Snail1/2 (115). In OC, FGF18 regulates OC cell migration, invasion and tumorigenicity through the NF-κB pathway, and increases the expression of cytokines IL-1A, IL-6, IL-8 and chemokines CXCL1 and CXCL2 (33). However, whether these pathways and secreted factors have regulatory effects in other tumors needs to be further explored and verified.

FGF18 mediates tumor progression by regulating the same signaling pathways in different cancers (**Figure 2**). For example, in CRC, HCC, EC, MPM and breast cancer, FGF18 regulates tumor cell or tissue progression through AKT and ERK signaling pathways after binding to FGFR (64, 79, 115, 143, 178); FGF18 promotes tumor progression through GSK3β pathway in CRC and breast cancer. Therefore, whether there are more common or different regulatory pathways of FGF18 in different tumors needs to be further explored. In addition, lncRNA H19-related mirnas were identified by differential non-coding RNA expression signatures and used as potential diagnostic biomarkers for GC and HCC (46, 47, 77). Besides these two cancers, it remains to be explored whether lncRNA H19-related mirnas can be used as diagnostic biomarkers in other tumors or whether there are other downstream miRNA targets to regulate tumor progression.

In addition, FGF18 is regulated by a variety of tumor autocrine or paracrine proteins (**Figure 2**). For example, in terms of steroid hormone regulation, progesterone induces the nuclear transcription factor heart and neural crest derivative expressed transcript 2 (HAND2) in endometrial stromal cells, leading to the reduction of FGF18 production in endometrial stromal cells. By reducing FGF18 levels, progesterone abolises stimulation of epithelial cell proliferation (200). Notably, BZA reduced FGF18 but did not affect HAND2, despite confirming the lower HAND2 mRNA expression in adenocarcinomas relative to normal tissues. Thus, it is different from progesterone regulation. FGF18 reduction is a potential mechanism by which BZA attenuates estrogen-induced endometrial proliferation and hyperplasia, revealing a novel mechanism for progestin-free treatment in postmenopausal women (36); overexpression of CD44, a membrane protein, significantly promotes tumor progression and is detrimental to chemotherapy prognosis (201–203), and the CD44 ^(+)^ subset of LT97 cells has the ability to prolong survival and growth in tumor cells, interestingly, FGF18 expression levels are elevated in this subset (68); Wnt/β-catenin is an important cancer targeting protein that plays an irreplaceable role in human cancers and experimental cancer models in animals (199, 204). Wnt/β-catenin signaling enhances FGF18 expression and promotes tumor progression in CRC, HCC, lung cancer, and breast cancer (32, 79, 115, 126). CD44 and Wnt/β-catenin may interact with FGF18 on the surface of tumor cell membrane to mediate the uptake of exogenous FGF18 by tumor cells through endocytosis and other cellular biological effects. As well as endocrine FGF18 regulates metabolism at the cell membrane to influence tumor progression. However, whether it has the same function in regulating metabolism in other cancers and how it directs tumor metabolism remain to be explored.

In addition, there are a variety of cellular or tissue metabolic substances that regulate tumor progression (**Figure 2**). For example, ALDH1 (high) cells exhibit cancer stem cell (CSC) properties such as phenotypic diversity and *in vivo* tumorigenicity, and FGF18 expression is elevated in the ALDH (high) subpopulation in OC (100), thus FGF18 is significantly correlated with cancer stem cell properties in OC; And FGF18 is a differentially expressed gene up-regulated by overexpression of HDACs in NSCLC. To date, therefore, the pathways by which FGF18 regulates tumor progression are very rich, the exact molecular mechanism of FGF18 in cancer is still unclear, and there are many more sites that still need to be explored, and in-depth study is expected to provide new therapeutic targets for these diseases.

## Author contributions

RZ designed the manuscript, YZ and TL drafted and wrote the manuscript, SS and YC designed the pictures and tables, and QY revised the manuscript.

## References

[1] C. Global Burden of Disease Cancer Kocarnik JM Compton K Dean FE Fu W Gaw BL. Mortality, years of life lost, years lived with disability, and disability-adjusted life years for 29 cancer groups from 2010 to 2019: a systematic analysis for the global burden of disease study 2019. JAMA Oncol (2022) 8:420–44. doi: 10.1001/jamaoncol.2021.6987 PMC8719276 34967848

[2] C. Global Burden of Disease Cancer Fitzmaurice C Akinyemiju TF Al Lami FH Alam T Alizadeh-Navaei R. Global, regional, and national cancer incidence, mortality, years of life lost, years lived with disability, and disability-adjusted life-years for 29 cancer groups, 1990 to 2016: a systematic analysis for the global burden of disease study. JAMA Oncol (2018) 4:1553–68. doi: 10.1001/jamaoncol.2018.2706 PMC6248091 29860482

[3] Fidler MM Gupta S Soerjomataram I Ferlay J Steliarova-Foucher E Bray F. Cancer incidence and mortality among young adults aged 20-39 years worldwide in 2012: a population-based study. Lancet Oncol (2017) 18:1579–89. doi: 10.1016/S1470-2045(17)30677-0 29111259

[4] Cao M Li H Sun D He S Yu Y Li J. Cancer screening in China: the current status, challenges, and suggestions. Cancer Lett (2021) 506:120–7. doi: 10.1016/j.canlet.2021.02.017 33684533

[5] Ferlay J Soerjomataram I Dikshit R Eser S Mathers C Rebelo M. Cancer incidence and mortality worldwide: sources, methods and major patterns in GLOBOCAN 2012. Int J Cancer (2015) 136:E359–86. doi: 10.1002/ijc.29210 25220842

[6] Sepich-Poore GD Zitvogel L Straussman R Hasty J Wargo JA Knight R. The microbiome and human cancer. Science (2021) 371(6536):eabc4552. doi: 10.1126/science.abc4552 33766858 PMC8767999

[7] Berger MF Mardis ER. The emerging clinical relevance of genomics in cancer medicine. Nat Rev Clin Oncol (2018) 15:353–65. doi: 10.1038/s41571-018-0002-6 PMC6658089 29599476

[8] Crowley E Di Nicolantonio F Loupakis F Bardelli A. Liquid biopsy: monitoring cancer-genetics in the blood. Nat Rev Clin Oncol (2013) 10:472–84. doi: 10.1038/nrclinonc.2013.110 23836314

[9] Gerlinger M Rowan AJ Horswell S Math M Larkin J Endesfelder D. Intratumor heterogeneity and branched evolution revealed by multiregion sequencing. N Engl J Med (2012) 366:883–92. doi: 10.1056/NEJMoa1113205 PMC4878653 22397650

[10] Katoh M. Multilayered prevention and treatment of chronic inflammation, organ fibrosis and cancer associated with canonical WNT/betacatenin signaling activation (Review). Int J Mol Med (2018) 42:713–25. doi: 10.3892/ijmm.2018.3689 PMC6034925 29786110

[11] Katoh M Katoh M. Comparative genomics on FGF8, FGF17, and FGF18 orthologs. Int J Mol Med (2005) 16:493–6. doi: 10.3892/ijmm.16.3.493 16077960

[12] Osawa T Tsuchida R Muramatsu M Shimamura T Wang F Suehiro J. Inhibition of histone demethylase JMJD1A improves anti-angiogenic therapy and reduces tumor-associated macrophages. Cancer Res (2013) 73:3019–28. doi: 10.1158/0008-5472.CAN-12-3231 23492365

[13] Buchtova M Chaloupkova R Zakrzewska M Vesela I Cela P Barathova J. Instability restricts signaling of multiple fibroblast growth factors. Cell Mol Life Sci (2015) 72:2445–59. doi: 10.1007/s00018-015-1856-8 PMC11113989 25854632

[14] Beenken A Mohammadi M. The FGF family: biology, pathophysiology and therapy. Nat Rev Drug Discovery (2009) 8:235–53. doi: 10.1038/nrd2792 PMC3684054 19247306

[15] Katoh M. Therapeutics targeting FGF signaling network in human diseases. Trends Pharmacol Sci (2016) 37:1081–96. doi: 10.1016/j.tips.2016.10.003 27992319

[16] Jing Q Wang Y Liu H Deng X Jiang L Liu R. FGFs: crucial factors that regulate tumour initiation and progression. Cell Prolif (2016) 49:438–47. doi: 10.1111/cpr.12275 PMC6496028 27383016

[17] Goetz R Ohnishi M Ding X Kurosu H Wang L Akiyoshi J. Klotho coreceptors inhibit signaling by paracrine fibroblast growth factor 8 subfamily ligands. Mol Cell Biol (2012) 32:1944–54. doi: 10.1128/MCB.06603-11 PMC3347405 22451487

[18] Tang D He Y Li W Li H. Wnt/beta-catenin interacts with the FGF pathway to promote proliferation and regenerative cell proliferation in the zebrafish lateral line neuromast. Exp Mol Med (2019) 51:1–16. doi: 10.1038/s12276-019-0247-x PMC6533250 31123246

[19] Turner N Grose R. Fibroblast growth factor signalling: from development to cancer. Nat Rev Cancer (2010) 10:116–29. doi: 10.1038/nrc2780 20094046

[20] Yu P Wilhelm K Dubrac A Tung JK Alves TC Fang JS. FGF-dependent metabolic control of vascular development. Nature (2017) 545:224–8. doi: 10.1038/nature22322 PMC5427179 28467822

[21] Porebska N Latko M Kucinska M Zakrzewska M Otlewski J Opalinski L. Targeting cellular trafficking of fibroblast growth factor receptors as a strategy for selective cancer treatment. J Clin Med (2018) 8(1):7. doi: 10.3390/jcm8010007 30577533 PMC6352210

[22] Tanner Y Grose RP. Dysregulated FGF signalling in neoplastic disorders. Semin Cell Dev Biol (2016) 53:126–35. doi: 10.1016/j.semcdb.2015.10.012 26463732

[23] Xie Y Su N Yang J Tan Q Huang S Jin M. FGF/FGFR signaling in health and disease. Signal Transduct Target Ther (2020) 5:181. doi: 10.1038/s41392-020-00222-7 32879300 PMC7468161

[24] Ohbayashi N Hoshikawa M Kimura S Yamasaki M Fukui S Itoh N. Structure and expression of the mRNA encoding a novel fibroblast growth factor, FGF-18. J Biol Chem (1998) 273:18161–4. doi: 10.1074/jbc.273.29.18161 9660775

[25] Savchenko E Teku GN Boza-Serrano A Russ K Berns M Deierborg T. FGF family members differentially regulate maturation and proliferation of stem cell-derived astrocytes. Sci Rep (2019) 9:9610. doi: 10.1038/s41598-019-46110-1 31270389 PMC6610107

[26] Xie Y Zinkle A Chen L Mohammadi M. Fibroblast growth factor signalling in osteoarthritis and cartilage repair. Nat Rev Rheumatol (2020) 16:547–64. doi: 10.1038/s41584-020-0469-2 32807927

[27] Tachmazidou I Hatzikotoulas K Southam L Esparza-Gordillo J Haberland V Zheng J. Identification of new therapeutic targets for osteoarthritis through genome-wide analyses of UK biobank data. Nat Genet (2019) 51:230–6. doi: 10.1038/s41588-018-0327-1 PMC6400267 30664745

[28] Shwartz Y Gonzalez-Celeiro M Chen CL Pasolli HA Sheu SH Fan SM. Cell types promoting goosebumps form a niche to regulate hair follicle stem cells. Cell (2020) 182:578–593 e19. doi: 10.1016/j.cell.2020.06.031 32679029 PMC7540726

[29] Goetz R Mohammadi M. Exploring mechanisms of FGF signalling through the lens of structural biology. Nat Rev Mol Cell Biol (2013) 14:166–80. doi: 10.1038/nrm3528 PMC3695728 23403721

[30] Latko M Czyrek A Porebska N Kucinska M Otlewski J Zakrzewska M. Cross-talk between fibroblast growth factor receptors and other cell surface proteins. Cells (2019) 8(5):455. doi: 10.3390/cells8050455 31091809 PMC6562592

[31] Hu MC Qiu WR Wang YP Hill D Ring BD Scully S. FGF-18, a novel member of the fibroblast growth factor family, stimulates hepatic and intestinal proliferation. Mol Cell Biol (1998) 18:6063–74. doi: 10.1128/MCB.18.10.6063 PMC109192 9742123

[32] Shimokawa T Furukawa Y Sakai M Li M Miwa N Lin YM. Involvement of the FGF18 gene in colorectal carcinogenesis, as a novel downstream target of the beta-catenin/T-cell factor complex. Cancer Res (2003) 63:6116–20. 14559787

[33] Wei W Mok SC Oliva E Kim SH Mohapatra G Birrer MJ. FGF18 as a prognostic and therapeutic biomarker in ovarian cancer. J Clin Invest (2013) 123:4435–48. doi: 10.1172/JCI70625 PMC3784549 24018557

[34] Yang L Yin D Wang Y Cao L. Inhibition of the growth of hepatocellular carcinoma cells through fibroblast growth factor 18 suppressed by miR-139. Oncol Rep (2017) 38:2565–71. doi: 10.3892/or.2017.5869 28765917

[35] Yu Z Lou L Zhao Y. Fibroblast growth factor 18 promotes the growth, migration and invasion of MDAMB231 cells. Oncol Rep (2018) 40:704–14. doi: 10.3892/or.2018.6482 PMC6072296 29901199

[36] Flannery CA Fleming AG Choe GH Naqvi H Zhang M Sharma A. Endometrial cancer-associated FGF18 expression is reduced by bazedoxifene in human endometrial stromal cells *In Vitro* and in murine endometrium. Endocrinology (2016) 157:3699–708. doi: 10.1210/en.2016-1233 PMC5045514 27267714

[37] Venerito M Nardone G Selgrad M Rokkas T Malfertheiner P. Gastric cancer–epidemiologic and clinical aspects. Helicobacter (2014) 19 Suppl 1:32–7. doi: 10.1111/hel.12164 25167943

[38] Razavi S Mahmoodi H Pandari HR Sarbakhsh P Shaghaghi A. Development and psychometric testing of a gastric cancer behavioural risk assessment inventory (GC-BRAI). East Mediterr Health J (2021) 27:50–8. doi: 10.26719/emhj.20.103 33538319

[39] Venerito M Link A Rokkas T Malfertheiner P. Gastric cancer - clinical and epidemiological aspects. Helicobacter (2016) 21 Suppl 1:39–44. doi: 10.1111/hel.12339 27531538

[40] Venerito M Vasapolli R Rokkas T Malfertheiner P. Gastric cancer: epidemiology, prevention, and therapy. Helicobacter (2018) 23 Suppl 1:e12518. doi: 10.1111/hel.12518 30203589

[41] Lansdorp-Vogelaar I Meester RGS Laszkowska M Escudero FA Ward ZJ Yeh JM. Cost-effectiveness of prevention and early detection of gastric cancer in Western countries. Best Pract Res Clin Gastroenterol (2021) 50-51:101735. doi: 10.1016/j.bpg.2021.101735 33975689

[42] Huang RJ Hwang JH. Improving the early diagnosis of gastric cancer. Gastrointest Endosc Clin N Am (2021) 31:503–17. doi: 10.1016/j.giec.2021.03.005 PMC8171812 34053636

[43] Zhang J Zhou Y Huang T Wu F Pan Y Dong Y. FGF18, a prominent player in FGF signaling, promotes gastric tumorigenesis through autocrine manner and is negatively regulated by miR-590-5p. Oncogene (2019) 38:33–46. doi: 10.1038/s41388-018-0430-x 30082912 PMC6318220

[44] Zhang J Tang PMK Zhou Y Cheng ASL Yu J Kang W. Targeting the oncogenic FGF-FGFR axis in gastric carcinogenesis. Cells (2019) 8(6):637. doi: 10.3390/cells8060637 31242658 PMC6627225

[45] Jomrich G Hudec X Harpain F Winkler D Timelthaler G Mohr T. Expression of FGF8, FGF18, and FGFR4 in gastroesophageal adenocarcinomas. Cells (2019) 8(9):1092. doi: 10.3390/cells8091092 31527546 PMC6770911

[46] Mohamed WA Schaalan MF Ramadan B. The expression profiling of circulating miR-204, miR-182, and lncRNA H19 as novel potential biomarkers for the progression of peptic ulcer to gastric cancer. J Cell Biochem (2019) 120:13464–77. doi: 10.1002/jcb.28620 30945348

[47] Schaalan M Mohamed W Fathy S. MiRNA-200c, MiRNA-139 and ln RNA H19; new predictors of treatment response in h-pylori- induced gastric ulcer or progression to gastric cancer. Microb Pathog (2020) 149:104442. doi: 10.1016/j.micpath.2020.104442 32795593

[48] Dienstmann R Rodon J Prat A Perez-Garcia J Adamo B Felip E. Genomic aberrations in the FGFR pathway: opportunities for targeted therapies in solid tumors. Ann Oncol (2014) 25:552–63. doi: 10.1093/annonc/mdt419 PMC4433501 24265351

[49] Park JM Han YM Oh JY Lee DY Choi SH Hahm KB. Transcriptome profiling implicated in beneficiary actions of kimchi extracts against helicobacter pylori infection. J Clin Biochem Nutr (2021) 69:171–87. doi: 10.3164/jcbn.20-116 PMC8482382 34616109

[50] Ghafouri-Fard S Esmaeili M Taheri M. H19 lncRNA: roles in tumorigenesis. BioMed Pharmacother (2020) 123:109774. doi: 10.1016/j.biopha.2019.109774 31855739

[51] Wang H Lu J Tang J Chen S He K Jiang X. Establishment of patient-derived gastric cancer xenografts: a useful tool for preclinical evaluation of targeted therapies involving alterations in HER-2, MET and FGFR2 signaling pathways. BMC Cancer (2017) 17:191. doi: 10.1186/s12885-017-3177-9 28292264 PMC5348902

[52] Aggarwal C Redman MW Lara PN Jr. Borghaei H Hoffman P Bradley JD. SWOG S1400D (NCT02965378), a phase II study of the fibroblast growth factor receptor inhibitor AZD4547 in previously treated patients with fibroblast growth factor pathway-activated stage IV squamous cell lung cancer (Lung-MAP substudy). J Thorac Oncol (2019) 14:1847–52. doi: 10.1016/j.jtho.2019.05.041 PMC6901020 31195180

[53] Zhang J Wong CC Leung KT Wu F Zhou Y Tong JHM. FGF18-FGFR2 signaling triggers the activation of c-Jun-YAP1 axis to promote carcinogenesis in a subgroup of gastric cancer patients and indicates translational potential. Oncogene (2020) 39:6647–63. doi: 10.1038/s41388-020-01458-x PMC7581496 32934314

[54] Force U.S.P.S.T. Davidson KW Barry MJ Mangione CM Cabana M Caughey AB. Screening for colorectal cancer: US preventive services task force recommendation statement. JAMA (2021) 325:1965–77. doi: 10.1001/jama.2021.6238 34003218

[55] Betesh AL Schnoll-Sussman FH. Colorectal cancer screening in the elderly. Clin Geriatr Med (2021) 37:173–83. doi: 10.1016/j.cger.2020.08.012 33213771

[56] Patel SG Karlitz JJ Yen T Lieu CH Boland CR. The rising tide of early-onset colorectal cancer: a comprehensive review of epidemiology, clinical features, biology, risk factors, prevention, and early detection. Lancet Gastroenterol Hepatol (2022) 7:262–74. doi: 10.1016/S2468-1253(21)00426-X 35090605

[57] Sivamaruthi BS Kesika P Chaiyasut C. The role of probiotics in colorectal cancer management. Evid Based Complement Alternat Med (2020) 2020:3535982. doi: 10.1155/2020/3535982 32148539 PMC7048916

[58] Li J Ma X Chakravarti D Shalapour S DePinho RA. Genetic and biological hallmarks of colorectal cancer. Genes Dev (2021) 35:787–820. doi: 10.1101/gad.348226.120 34074695 PMC8168558

[59] Xu K He J Zhang J Liu T Yang F Ren T. A novel prognostic risk score model based on immune-related genes in patients with stage IV colorectal cancer. Biosci Rep (2020) 40(10):BSR20201725. doi: 10.1042/BSR20201725 33034614 PMC7584813

[60] Wu B Yang J Qin Z Yang H Shao J Shang Y. Prognosis prediction of stage IV colorectal cancer patients by mRNA transcriptional profile. Cancer Med (2022) 11(24):4900–12. doi: 10.1002/cam4.4824 PMC9761091 35587572

[61] Bardou M Barkun AN Martel M. Obesity and colorectal cancer. Gut (2013) 62:933–47. doi: 10.1136/gutjnl-2013-304701 23481261

[62] Soltani G Poursheikhani A Yassi M Hayatbakhsh A Kerachian M Kerachian MA. Obesity, diabetes and the risk of colorectal adenoma and cancer. BMC Endocr Disord (2019) 19:113. doi: 10.1186/s12902-019-0444-6 31664994 PMC6819551

[63] Campanella G Gunter MJ Polidoro S Krogh V Palli D Panico S. Epigenome-wide association study of adiposity and future risk of obesity-related diseases. Int J Obes (Lond) (2018) 42:2022–35. doi: 10.1038/s41366-018-0064-7 29713043

[64] Sonvilla G Allerstorfer S Stattner S Karner J Klimpfinger M Fischer H. FGF18 in colorectal tumour cells: autocrine and paracrine effects. Carcinogenesis (2008) 29:15–24. doi: 10.1093/carcin/bgm202 17890768

[65] Teimoori-Toolabi L Azadmanesh K Zeinali S. Selective suicide gene therapy of colon cancer cell lines exploiting fibroblast growth factor 18 promoter. Cancer Biother Radiopharm (2010) 25:105–16. doi: 10.1089/cbr.2009.0643 20187803

[66] Erdem ZN Schwarz S Drev D Heinzle C Reti A Heffeter P. Irinotecan upregulates fibroblast growth factor receptor 3 expression in colorectal cancer cells, which mitigates irinotecan-induced apoptosis. Transl Oncol (2017) 10:332–9. doi: 10.1016/j.tranon.2017.02.004 PMC5367848 28340475

[67] Sonvilla G Allerstorfer S Heinzle C Stattner S Karner J Klimpfinger M. Fibroblast growth factor receptor 3-IIIc mediates colorectal cancer growth and migration. Br J Cancer (2010) 102:1145–56. doi: 10.1038/sj.bjc.6605596 PMC2853090 20234367

[68] Koneczny I Schulenburg A Hudec X Knofler M Holzmann K Piazza G. Autocrine fibroblast growth factor 18 signaling mediates wnt-dependent stimulation of CD44-positive human colorectal adenoma cells. Mol Carcinog (2015) 54:789–99. doi: 10.1002/mc.22146 PMC4162857 24619956

[69] Katoh M Katoh M. NUMB is a break of WNT-notch signaling cycle. Int J Mol Med (2006) 18:517–21. doi: 10.3892/ijmm.18.3.517 16865239

[70] Katoh M Katoh M. Cross-talk of WNT and FGF signaling pathways at GSK3beta to regulate beta-catenin and SNAIL signaling cascades. Cancer Biol Ther (2006) 5:1059–64. doi: 10.4161/cbt.5.9.3151 16940750

[71] Li X Ramadori P Pfister D Seehawer M Zender L Heikenwalder M. The immunological and metabolic landscape in primary and metastatic liver cancer. Nat Rev Cancer (2021) 21:541–57. doi: 10.1038/s41568-021-00383-9 34326518

[72] Llovet JM Kelley RK Villanueva A Singal AG Pikarsky E Roayaie S. Hepatocellular carcinoma. Nat Rev Dis Primers (2021) 7:6. doi: 10.1038/s41572-020-00240-3 33479224 PMC13537433

[73] Orcutt ST Anaya DA. Liver resection and surgical strategies for management of primary liver cancer. Cancer Control (2018) 25:1073274817744621. doi: 10.1177/1073274817744621 29327594 PMC5933574

[74] Estes C Razavi H Loomba R Younossi Z Sanyal AJ. Modeling the epidemic of nonalcoholic fatty liver disease demonstrates an exponential increase in burden of disease. Hepatology (2018) 67:123–33. doi: 10.1002/hep.29466 PMC5767767 28802062

[75] Anwanwan D Singh SK Singh S Saikam V Singh R. Challenges in liver cancer and possible treatment approaches. Biochim Biophys Acta Rev Cancer (2020) 1873:188314. doi: 10.1016/j.bbcan.2019.188314 31682895 PMC6981221

[76] Gauglhofer C Sagmeister S Schrottmaier W Fischer C Rodgarkia-Dara C Mohr T. Up-regulation of the fibroblast growth factor 8 subfamily in human hepatocellular carcinoma for cell survival and neoangiogenesis. Hepatology (2011) 53:854–64. doi: 10.1002/hep.24099 21319186

[77] Matouk IJ DeGroot N Mezan S Ayesh S Abu-lail R Hochberg A. The H19 non-coding RNA is essential for human tumor growth. PloS One (2007) 2:e845. doi: 10.1371/journal.pone.0000845 17786216 PMC1959184

[78] Turner NC Slamon DJ Ro J Bondarenko I Im SA Masuda N. Overall survival with palbociclib and fulvestrant in advanced breast cancer. N Engl J Med (2018) 379:1926–36. doi: 10.1056/NEJMoa1810527 30345905

[79] Guo P Wang Y Dai C Tao C Wu F Xie X. Ribosomal protein S15a promotes tumor angiogenesis *via* enhancing wnt/beta-catenin-induced FGF18 expression in hepatocellular carcinoma. Oncogene (2018) 37:1220–36. doi: 10.1038/s41388-017-0017-y 29242604

[80] Armstrong DK Alvarez RD Bakkum-Gamez JN Barroilhet L Behbakht K Berchuck A. Ovarian cancer, version 2.2020, NCCN clinical practice guidelines in oncology. J Natl Compr Canc Netw (2021) 19:191–226. doi: 10.6004/jnccn.2021.0007 33545690

[81] Torre LA Trabert B DeSantis CE Miller KD Samimi G Runowicz CD. Ovarian cancer statistics, 2018. CA Cancer J Clin (2018) 68:284–96. doi: 10.3322/caac.21456 PMC6621554 29809280

[82] Menon U Karpinskyj C Gentry-Maharaj A. Ovarian cancer prevention and screening. Obstet Gynecol (2018) 131:909–27. doi: 10.1097/AOG.0000000000002580 29630008

[83] Menon U Gentry-Maharaj A Burnell M Singh N Ryan A Karpinskyj C. Ovarian cancer population screening and mortality after long-term follow-up in the UK collaborative trial of ovarian cancer screening (UKCTOCS): a randomised controlled trial. Lancet (2021) 397:2182–93. doi: 10.1016/S0140-6736(21)00731-5 PMC8192829 33991479

[84] Kossai M Leary A Scoazec JY Genestie C. Ovarian cancer: a heterogeneous disease. Pathobiology (2018) 85:41–9. doi: 10.1159/000479006 29020678

[85] Vathipadiekal V Wang V Wei W Waldron L Drapkin R Gillette M. Creation of a human secretome: a novel composite library of human secreted proteins: validation using ovarian cancer gene expression data and a virtual secretome array. Clin Cancer Res (2015) 21:4960–9. doi: 10.1158/1078-0432.CCR-14-3173 25944803

[86] Peng X Yu M Chen J. Transcriptome sequencing identifies genes associated with invasion of ovarian cancer. J Int Med Res (2020) 48:300060520950912. doi: 10.1177/0300060520950912 32878513 PMC7780583

[87] Kulbe H Otto R Darb-Esfahani S Lammert H Abobaker S Welsch G. Discovery and validation of novel biomarkers for detection of epithelial ovarian cancer. Cells (2019) 8(7):713. doi: 10.3390/cells8070713 31336942 PMC6678810

[88] El-Gendi S Abdelzaher E Mostafa MF Sheasha GA. FGF18 as a potential biomarker in serous and mucinous ovarian tumors. Tumour Biol (2016) 37:3173–83. doi: 10.1007/s13277-015-4129-0 26427667

[89] Tang M Wang T Zhang X. A review of SNP heritability estimation methods. Brief Bioinform (2022) 23(3):bbac067. doi: 10.1093/bib/bbac067 35289357

[90] Meng QH Xu E Hildebrandt MA Liang D Lu K Ye Y. Genetic variants in the fibroblast growth factor pathway as potential markers of ovarian cancer risk, therapeutic response, and clinical outcome. Clin Chem (2014) 60:222–32. doi: 10.1373/clinchem.2013.211490 PMC4437198 24146310

[91] Shi K Yin X Cai MC Yan Y Jia C Ma P. PAX8 regulon in human ovarian cancer links lineage dependency with epigenetic vulnerability to HDAC inhibitors. Elife (2019) 8:e44306. doi: 10.7554/eLife.44306 31050342 PMC6533083

[92] Chang B Liu G Xue F Rosen DG Xiao L Wang X. ALDH1 expression correlates with favorable prognosis in ovarian cancers. Mod Pathol (2009) 22:817–23. doi: 10.1038/modpathol.2009.35 PMC2692456 19329942

[93] Landen CN Jr. Goodman B Katre AA Steg AD Nick AM Stone RL. Targeting aldehyde dehydrogenase cancer stem cells in ovarian cancer. Mol Cancer Ther (2010) 9:3186–99. doi: 10.1158/1535-7163.MCT-10-0563 PMC3005138 20889728

[94] Silva IA Bai S McLean K Yang K Griffith K Thomas D. Aldehyde dehydrogenase in combination with CD133 defines angiogenic ovarian cancer stem cells that portend poor patient survival. Cancer Res (2011) 71:3991–4001. doi: 10.1158/0008-5472.CAN-10-3175 21498635 PMC3107359

[95] Ma I Allan AL. The role of human aldehyde dehydrogenase in normal and cancer stem cells. Stem Cell Rev Rep (2011) 7:292–306. doi: 10.1007/s12015-010-9208-4 21103958

[96] Nassar D Blanpain C. Cancer stem cells: basic concepts and therapeutic implications. Annu Rev Pathol (2016) 11:47–76. doi: 10.1146/annurev-pathol-012615-044438 27193450

[97] Ghiaur G Gerber J Jones RJ. Concise review: cancer stem cells and minimal residual disease. Stem Cells (2012) 30:89–93. doi: 10.1002/stem.769 22045578 PMC4685474

[98] Bapat SA Mali AM Koppikar CB Kurrey NK. Stem and progenitor-like cells contribute to the aggressive behavior of human epithelial ovarian cancer. Cancer Res (2005) 65:3025–9. doi: 10.1158/0008-5472.CAN-04-3931 15833827

[99] Gao MQ Choi YP Kang S Youn JH Cho NH. CD24+ cells from hierarchically organized ovarian cancer are enriched in cancer stem cells. Oncogene (2010) 29:2672–80. doi: 10.1038/onc.2010.35 20190812

[100] Sharrow AC Perkins B Collector MI Yu W Simons BW Jones RJ. Characterization of aldehyde dehydrogenase 1 high ovarian cancer cells: towards targeted stem cell therapy. Gynecol Oncol (2016) 142:341–8. doi: 10.1016/j.ygyno.2016.03.022 PMC5843190 27017984

[101] Loibl S Poortmans P Morrow M Denkert C Curigliano G. Breast cancer. Lancet (2021) 397:1750–69. doi: 10.1016/S0140-6736(20)32381-3 33812473

[102] Sung H Ferlay J Siegel RL Laversanne M Soerjomataram I Jemal A. Global cancer statistics 2020: GLOBOCAN estimates of incidence and mortality worldwide for 36 cancers in 185 countries. CA Cancer J Clin (2021) 71:209–49. doi: 10.3322/caac.21660 33538338

[103] Hereditary cancer syndromes and risk assessment: ACOG COMMITTEE OPINION SUMMARY, number 793. Obstet Gynecol (2019) 134:1366–7. doi: 10.1097/AOG.0000000000003563 31764755

[104] Forbes C Fayter D de Kock S Quek RG. A systematic review of international guidelines and recommendations for the genetic screening, diagnosis, genetic counseling, and treatment of BRCA-mutated breast cancer. Cancer Manag Res (2019) 11:2321–37. doi: 10.2147/CMAR.S189627 PMC6434912 30962720

[105] C. Collaborative Group on Hormonal Factors in Breast. Type and timing of menopausal hormone therapy and breast cancer risk: individual participant meta-analysis of the worldwide epidemiological evidence. Lancet (2019) 394:1159–68. doi: 10.1016/S0140-6736(19)31709-X PMC6891893 31474332

[106] Oeffinger KC Fontham ET Etzioni R Herzig A Michaelson JS Shih YC. Breast cancer screening for women at average risk: 2015 guideline update from the American cancer society. JAMA (2015) 314:1599–614. doi: 10.1001/jama.2015.12783 PMC4831582 26501536

[107] Morch LS Skovlund CW Hannaford PC Iversen L Fielding S Lidegaard O. Contemporary hormonal contraception and the risk of breast cancer. N Engl J Med (2017) 377:2228–39. doi: 10.1056/NEJMoa1700732 29211679

[108] Trayes KP Cokenakes SEH. Breast cancer treatment. Am Fam Physician (2021) 104:171–8. 34383430

[109] Waks AG Winer EP. Breast cancer treatment: a review. JAMA (2019) 321:288–300. doi: 10.1001/jama.2018.19323 30667505

[110] Gao JJ Cheng J Bloomquist E Sanchez J Wedam SB Singh H. CDK4/6 inhibitor treatment for patients with hormone receptor-positive, HER2-negative, advanced or metastatic breast cancer: a US food and drug administration pooled analysis. Lancet Oncol (2020) 21:250–60. doi: 10.1016/S1470-2045(19)30804-6 31859246

[111] Harbeck N Gnant M. Breast cancer. Lancet (2017) 389:1134–50. doi: 10.1016/S0140-6736(16)31891-8 27865536

[112] Picon-Ruiz M Morata-Tarifa C Valle-Goffin JJ Friedman ER Slingerland JM. Obesity and adverse breast cancer risk and outcome: mechanistic insights and strategies for intervention. CA Cancer J Clin (2017) 67:378–97. doi: 10.3322/caac.21405 PMC5591063 28763097

[113] Fischer BE Will H. Effects of intact fibrin and partially plasmin-degraded fibrin on kinetic properties of one-chain tissue-type plasminogen activator. Biochim Biophys Acta (1990) 1041:48–54. doi: 10.1016/0167-4838(90)90121-U 2145980

[114] Mustacchi G Sormani MP Bruzzi P Gennari A Zanconati F Bonifacio D. Identification and validation of a new set of five genes for prediction of risk in early breast cancer. Int J Mol Sci (2013) 14:9686–702. doi: 10.3390/ijms14059686 PMC3676806 23648477

[115] Song N Zhong J Hu Q Gu T Yang B Zhang J. FGF18 enhances migration and the epithelial-mesenchymal transition in breast cancer by regulating Akt/GSK3beta/Beta-catenin signaling. Cell Physiol Biochem (2018) 49:1019–32. doi: 10.1159/000493286 30196303

[116] Cancer of the lung. JAMA (2021) 325:1010. doi: 10.1001/jama.2020.17834 33687456

[117] Force U.S.P.S.T. Krist AH Davidson KW Mangione CM Barry MJ Cabana M. Screening for lung cancer: US preventive services task force recommendation statement. JAMA (2021) 325:962–70. doi: 10.1001/jama.2021.1117 33687470

[118] Bracken-Clarke D Kapoor D Baird AM Buchanan PJ Gately K Cuffe S. Vaping and lung cancer - a review of current data and recommendations. Lung Cancer (2021) 153:11–20. doi: 10.1016/j.lungcan.2020.12.030 33429159

[119] Tringali G Milanese G Ledda RE Pastorino U Sverzellati N Silva M. Lung cancer screening: evidence, risks, and opportunities for implementation. Rofo (2021) 193:1153–61. doi: 10.1055/a-1382-8648 33772489

[120] Jin J. Screening for lung cancer. JAMA (2021) 325:1016. doi: 10.1001/jama.2021.1799 33687464

[121] Hirsch FR Scagliotti GV Mulshine JL Kwon R Curran WJ Jr. Wu YL. Lung cancer: current therapies and new targeted treatments. Lancet (2017) 389:299–311. doi: 10.1016/S0140-6736(16)30958-8 27574741

[122] Salazar MC Rosen JE Wang Z Arnold BN Thomas DC Herbst RS. Association of delayed adjuvant chemotherapy with survival after lung cancer surgery. JAMA Oncol (2017) 3:610–9. doi: 10.1001/jamaoncol.2016.5829 PMC5824207 28056112

[123] Kadara H Tran LM Liu B Vachani A Li S Sinjab A. Early diagnosis and screening for lung cancer. Cold Spring Harb Perspect Med (2021) 11(9):a037994. doi: 10.1101/cshperspect.a037994 34001525 PMC8415293

[124] Li G Tian Y Zhu WG. The roles of histone deacetylases and their inhibitors in cancer therapy. Front Cell Dev Biol (2020) 8:576946. doi: 10.3389/fcell.2020.576946 33117804 PMC7552186

[125] Parra M. Class IIa HDACs - new insights into their functions in physiology and pathology. FEBS J (2015) 282:1736–44. doi: 10.1111/febs.13061 25244360

[126] Guo K Ma Z Zhang Y Han L Shao C Feng Y. HDAC7 promotes NSCLC proliferation and metastasis *via* stabilization by deubiquitinase USP10 and activation of beta-catenin-FGF18 pathway. J Exp Clin Cancer Res (2022) 41:91. doi: 10.1186/s13046-022-02266-9 35277183 PMC8915541

[127] Chen T Gong W Tian H Wang H Chu S Ma J. Fibroblast growth factor 18 promotes proliferation and migration of H460 cells *via* the ERK and p38 signaling pathways. Oncol Rep (2017) 37:1235–42. doi: 10.3892/or.2016.5301 27959447

[128] Dutt A Ramos AH Hammerman PS Mermel C Cho J Sharifnia T. Inhibitor-sensitive FGFR1 amplification in human non-small cell lung cancer. PloS One (2011) 6:e20351. doi: 10.1371/journal.pone.0020351 21666749 PMC3110189

[129] Harding TC Long L Palencia S Zhang H Sadra A Hestir K. Blockade of nonhormonal fibroblast growth factors by FP-1039 inhibits growth of multiple types of cancer. Sci Transl Med (2013) 5:178ra39. doi: 10.1126/scitranslmed.3005414 23536011

[130] Murali R Delair DF Bean SM Abu-Rustum NR Soslow RA. Evolving roles of histologic evaluation and Molecular/Genomic profiling in the management of endometrial cancer. J Natl Compr Canc Netw (2018) 16:201–9. doi: 10.6004/jnccn.2017.7066 PMC6639790 29439179

[131] Ferlay J Colombet M Soerjomataram I Mathers C Parkin DM Pineros M. Estimating the global cancer incidence and mortality in 2018: GLOBOCAN sources and methods. Int J Cancer (2019) 144:1941–53. doi: 10.1002/ijc.31937 30350310

[132] Brooks RA Fleming GF Lastra RR Lee NK Moroney JW Son CH. Current recommendations and recent progress in endometrial cancer. CA Cancer J Clin (2019) 69:258–79. doi: 10.3322/caac.21561 31074865

[133] Clarke MA Long BJ Del Mar Morillo A Arbyn M Bakkum-Gamez JN Wentzensen N. Association of endometrial cancer risk with postmenopausal bleeding in women: a systematic review and meta-analysis. JAMA Intern Med (2018) 178:1210–22. doi: 10.1001/jamainternmed.2018.2820 PMC6142981 30083701

[134] Hazelwood E Sanderson E Tan VY Ruth KS Frayling TM Dimou N. Identifying molecular mediators of the relationship between body mass index and endometrial cancer risk: a mendelian randomization analysis. BMC Med (2022) 20:125. doi: 10.1186/s12916-022-02322-3 35436960 PMC9017004

[135] Morice P Leary A Creutzberg C Abu-Rustum N Darai E. Endometrial cancer. Lancet (2016) 387:1094–108. doi: 10.1016/S0140-6736(15)00130-0 26354523

[136] Onstad MA Schmandt RE Lu KH. Addressing the role of obesity in endometrial cancer risk, prevention, and treatment. J Clin Oncol (2016) 34:4225–30. doi: 10.1200/JCO.2016.69.4638 PMC5455320 27903150

[137] Morrison J Balega J Buckley L Clamp A Crosbie E Drew Y. British Gynaecological cancer society (BGCS) uterine cancer guidelines: recommendations for practice. Eur J Obstet Gynecol Reprod Biol (2022) 270:50–89. doi: 10.1016/j.ejogrb.2021.11.423 35065448

[138] Galaal K Donkers H Bryant A Lopes AD. Laparoscopy versus laparotomy for the management of early stage endometrial cancer. Cochrane Database Syst Rev (2018) 10:CD006655. doi: 10.1002/14651858.CD006655.pub3 30379327 PMC6517108

[139] Walker JL Piedmonte MR Spirtos NM Eisenkop SM Schlaerth JB Mannel RS. Recurrence and survival after random assignment to laparoscopy versus laparotomy for comprehensive surgical staging of uterine cancer: gynecologic oncology group LAP2 study. J Clin Oncol (2012) 30:695–700. doi: 10.1200/JCO.2011.38.8645 22291074 PMC3295548

[140] Janda M Gebski V Davies LC Forder P Brand A Hogg R. Effect of total laparoscopic hysterectomy vs total abdominal hysterectomy on disease-free survival among women with stage I endometrial cancer: a randomized clinical trial. JAMA (2017) 317:1224–33. doi: 10.1001/jama.2017.2068 28350928

[141] Crosbie EJ Kitson SJ McAlpine JN Mukhopadhyay A Powell ME Singh N. Endometrial cancer. Lancet (2022) 399:1412–28. doi: 10.1016/S0140-6736(22)00323-3 35397864

[142] Estienne A Price CA. The fibroblast growth factor 8 family in the female reproductive tract. Reproduction (2018) 155:R53–62. doi: 10.1530/REP-17-0542 29269444

[143] Wu J Tao X Zhang H Yi XH Yu YH. Estrogen-induced stromal FGF18 promotes proliferation and invasion of endometrial carcinoma cells through ERK and akt signaling. Cancer Manag Res (2020) 12:6767–77. doi: 10.2147/CMAR.S254242 PMC7414926 32801905

[144] Ono Y Yoshino O Hiraoka T Sato E Fukui Y Ushijima A. CD206+ M2-like macrophages are essential for successful implantation. Front Immunol (2020) 11:557184. doi: 10.3389/fimmu.2020.557184 33193326 PMC7644510

[145] Turajlic S Swanton C Boshoff C. Kidney cancer: the next decade. J Exp Med (2018) 215:2477–9. doi: 10.1084/jem.20181617 PMC6170181 30217855

[146] Linehan WM Schmidt LS Crooks DR Wei D Srinivasan R Lang M. The metabolic basis of kidney cancer. Cancer Discovery (2019) 9:1006–21. doi: 10.1158/2159-8290.CD-18-1354 31088840

[147] Owens B. Kidney cancer. Nature (2016) 537:S97. doi: 10.1038/537S97a 27626782

[148] Chowdhury N Drake CG. Kidney cancer: an overview of current therapeutic approaches. Urol Clin North Am (2020) 47:419–31. doi: 10.1016/j.ucl.2020.07.009 33008493

[149] Yang C Zhang Z Ye F Mou Z Chen X Ou Y. FGF18 inhibits clear cell renal cell carcinoma proliferation and invasion *via* regulating epithelial-mesenchymal transition. Front Oncol (2020) 10:1685. doi: 10.3389/fonc.2020.01685 33117668 PMC7552945

[150] Stacchiotti S Van Tine BA. Synovial sarcoma: current concepts and future perspectives. J Clin Oncol (2018) 36:180–7. doi: 10.1200/JCO.2017.75.1941 29220290

[151] Mastrangelo G Coindre JM Ducimetiere F Dei Tos AP Fadda E Blay JY. Incidence of soft tissue sarcoma and beyond: a population-based prospective study in 3 European regions. Cancer (2012) 118:5339–48. doi: 10.1002/cncr.27555 22517534

[152] Gazendam AM Popovic S Munir S Parasu N Wilson D Ghert M. Synovial sarcoma: a clinical review. Curr Oncol (2021) 28:1909–20. doi: 10.3390/curroncol28030177 PMC8161765 34069748

[153] Noujaim J Constantinidou A Messiou C Thway K Miah A Benson C. Successful ifosfamide rechallenge in soft-tissue sarcoma. Am J Clin Oncol (2018) 41:147–51. doi: 10.1097/COC.0000000000000243 26523439

[154] Ferrari A De Salvo GL Brennan B van Noesel MM De Paoli A Casanova M. Synovial sarcoma in children and adolescents: the European pediatric soft tissue sarcoma study group prospective trial (EpSSG NRSTS 2005). Ann Oncol (2015) 26:567–72. doi: 10.1093/annonc/mdu562 25488687

[155] Vlenterie M Ho VK Kaal SE Vlenterie R Haas R van der Graaf WT. Age as an independent prognostic factor for survival of localised synovial sarcoma patients. Br J Cancer (2015) 113:1602–6. doi: 10.1038/bjc.2015.375 PMC4705887 26554650

[156] Ishibe T Nakayama T Okamoto T Aoyama T Nishijo K Shibata KR. Disruption of fibroblast growth factor signal pathway inhibits the growth of synovial sarcomas: potential application of signal inhibitors to molecular target therapy. Clin Cancer Res (2005) 11:2702–12. doi: 10.1158/1078-0432.CCR-04-2057 15814652

[157] Grand EK Chase AJ Heath C Rahemtulla A Cross NC. Targeting FGFR3 in multiple myeloma: inhibition of t(4;14)-positive cells by SU5402 and PD173074. Leukemia (2004) 18:962–6. doi: 10.1038/sj.leu.2403347 15029211

[158] Gudernova I Vesela I Balek L Buchtova M Dosedelova H Kunova M. Multikinase activity of fibroblast growth factor receptor (FGFR) inhibitors SU5402, PD173074, AZD1480, AZD4547 and BGJ398 compromises the use of small chemicals targeting FGFR catalytic activity for therapy of short-stature syndromes. Hum Mol Genet (2016) 25:9–23. doi: 10.1093/hmg/ddv441 26494904

[159] Calandrella N Risuleo G Scarsella G Mustazza C Castelli M Galati F. Reduction of cell proliferation induced by PD166866: an inhibitor of the basic fibroblast growth factor. J Exp Clin Cancer Res (2007) 26:405–9. 17987803

[160] Risuleo G Ciacciarelli M Castelli M Galati G. The synthetic inhibitor of fibroblast growth factor receptor PD166866 controls negatively the growth of tumor cells in culture. J Exp Clin Cancer Res (2009) 28:151. doi: 10.1186/1756-9966-28-151 20003343 PMC2797793

[161] Wang Y Shen N Li S Yu H Wang Y Liu Z. Synergistic therapy for cervical cancer by codelivery of cisplatin and JQ1 inhibiting Plk1-mutant Trp53 axis. Nano Lett (2021) 21:2412–21. doi: 10.1021/acs.nanolett.0c04402 33705152

[162] Zhang L Jiang Y Lu X Zhao H Chen C Wang Y. Genomic characterization of cervical cancer based on human papillomavirus status. Gynecol Oncol (2019) 152:629–37. doi: 10.1016/j.ygyno.2018.12.017 30581036

[163] Pan BS Wang YK Lai MS Mu YF Huang BM. Cordycepin induced MA-10 mouse leydig tumor cell apoptosis by regulating p38 MAPKs and PI3K/AKT signaling pathways. Sci Rep (2015) 5:13372. doi: 10.1038/srep13372 26303320 PMC4548195

[164] Fankhauser CD Grogg JB Hayoz S Wettstein MS Dieckmann KP Sulser T. Risk factors and treatment outcomes of 1,375 patients with testicular leydig cell tumors: analysis of published case series data. J Urol (2020) 203:949–56. doi: 10.1097/JU.0000000000000705 31845841

[165] Yoon SY Park SJ Park YJ. The anticancer properties of cordycepin and their underlying mechanisms. Int J Mol Sci (2018) 19(10):3027. doi: 10.3390/ijms19103027 30287757 PMC6212910

[166] Nakamura K Shinozuka K Yoshikawa N. Anticancer and antimetastatic effects of cordycepin, an active component of cordyceps sinensis. J Pharmacol Sci (2015) 127:53–6. doi: 10.1016/j.jphs.2014.09.001 25704018

[167] Chang MM Hong SY Yang SH Wu CC Wang CY Huang BM. Anti-cancer effect of cordycepin on FGF9-induced testicular tumorigenesis. Int J Mol Sci (2020) 21(21):8336. doi: 10.3390/ijms21218336 33172093 PMC7672634

[168] Zhou L Cheng X Connolly BA Dickman MJ Hurd PJ Hornby DP. Zebularine: a novel DNA methylation inhibitor that forms a covalent complex with DNA methyltransferases. J Mol Biol (2002) 321:591–9. doi: 10.1016/S0022-2836(02)00676-9 PMC2713825 12206775

[169] Cheng JC Weisenberger DJ Gonzales FA Liang G Xu GL Hu YG. Continuous zebularine treatment effectively sustains demethylation in human bladder cancer cells. Mol Cell Biol (2004) 24:1270–8. doi: 10.1128/MCB.24.3.1270-1278.2004 PMC321446 14729971

[170] Christoph F Kempkensteffen C Weikert S Kollermann J Krause H Miller K. Methylation of tumour suppressor genes APAF-1 and DAPK-1 and *in vitro* effects of demethylating agents in bladder and kidney cancer. Br J Cancer (2006) 95:1701–7. doi: 10.1038/sj.bjc.6603482 PMC2360762 17133271

[171] Veerla S Panagopoulos I Jin Y Lindgren D Hoglund M. Promoter analysis of epigenetically controlled genes in bladder cancer. Genes Chromosomes Cancer (2008) 47:368–78. doi: 10.1002/gcc.20542 18196590

[172] Crona J Taieb D Pacak K. New perspectives on pheochromocytoma and paraganglioma: toward a molecular classification. Endocr Rev (2017) 38:489–515. doi: 10.1210/er.2017-00062 28938417 PMC5716829

[173] Meijs AC Snel M Corssmit EPM. Pheochromocytoma/paraganglioma crisis: case series from a tertiary referral center for pheochromocytomas and paragangliomas. Hormones (Athens) (2021) 20:395–403. doi: 10.1007/s42000-021-00274-6 33575936 PMC8110488

[174] Wachtel H Fishbein L. Genetics of pheochromocytoma and paraganglioma. Curr Opin Endocrinol Diabetes Obes (2021) 28:283–90. doi: 10.1097/MED.0000000000000634 33764930

[175] Suh YJ Choe JY Park HJ. Malignancy in pheochromocytoma or paraganglioma: integrative analysis of 176 cases in TCGA. Endocr Pathol (2017) 28:159–64. doi: 10.1007/s12022-017-9479-2 28386672

[176] Baas P Scherpereel A Nowak AK Fujimoto N Peters S Tsao AS. First-line nivolumab plus ipilimumab in unresectable malignant pleural mesothelioma (CheckMate 743): a multicentre, randomised, open-label, phase 3 trial. Lancet (2021) 397:375–86. doi: 10.1016/S0140-6736(20)32714-8 33485464

[177] Simon M Shochat T Peled N Zer A Kramer MR Eldan O. Intensity-modulated radiotherapy is a safe and effective treatment for localized malignant pleural mesothelioma. Thorac Cancer (2018) 9:1470–5. doi: 10.1111/1759-7714.12860 PMC6209784 30240138

[178] Schelch K Hoda MA Klikovits T Munzker J Ghanim B Wagner C. Fibroblast growth factor receptor inhibition is active against mesothelioma and synergizes with radio- and chemotherapy. Am J Respir Crit Care Med (2014) 190:763–72. doi: 10.1164/rccm.201404-0658OC 25188816

[179] Marzagalli M Ebelt ND Manuel ER. Unraveling the crosstalk between melanoma and immune cells in the tumor microenvironment. Semin Cancer Biol (2019) 59:236–50. doi: 10.1016/j.semcancer.2019.08.002 31404607

[180] Merkel EA Mohan LS Shi K Panah E Zhang B Gerami P. Paediatric melanoma: clinical update, genetic basis, and advances in diagnosis. Lancet Child Adolesc Health (2019) 3:646–54. doi: 10.1016/S2352-4642(19)30116-6 31204309

[181] Metzner T Bedeir A Held G Peter-Vorosmarty B Ghassemi S Heinzle C. Fibroblast growth factor receptors as therapeutic targets in human melanoma: synergism with BRAF inhibition. J Invest Dermatol (2011) 131:2087–95. doi: 10.1038/jid.2011.177 PMC3383623 21753785

[182] Motzer RJ Dmitrovsky E Miller WH Jr. Tong WP Bajorin DF Scher HI. Suramin for germ cell tumors. *In vitro* growth inhibition and results of a phase II trial. Cancer (1993) 72:3313–7. doi: 10.1002/1097-0142(19931201)72:11<3313::AID-CNCR2820721129>3.0.CO;2-C 8242558

[183] Granerus M Engstrom W. Dual effects of four members of the fibroblast growth factor member family on multiplication and motility in human teratocarcinoma cells *in vitro* . Anticancer Res (2000) 20:3527–31. doi: 10.1243/09544054JEM325 11131657

[184] Mishra A Jaiswal R Amita P Mishra SC. Molecular interactions in juvenile nasopharyngeal angiofibroma: preliminary signature and relevant review. Eur Arch Otorhinolaryngol (2019) 276:93–100. doi: 10.1007/s00405-018-5178-y 30387011

[185] Pool C Gates CJ Patel VA Carr MM. Juvenile nasopharyngeal angiofibroma: national practice patterns and resource utilization *via* HCUP KID. Int J Pediatr Otorhinolaryngol (2021) 149:110871. doi: 10.1016/j.ijporl.2021.110871 34385042

[186] Silveira SM Custodio Domingues MA Butugan O Brentani MM Rogatto SR. Tumor microenvironmental genomic alterations in juvenile nasopharyngeal angiofibroma. Head Neck (2012) 34:485–92. doi: 10.1002/hed.21767 22489321

[187] Perez-Ramirez M Hernandez-Jimenez AJ Guerrero-Guerrero A Benadon-Darszon E Perezpena-Diazconti M Siordia-Reyes AG. Genomics and epigenetics: a study of ependymomas in pediatric patients. Clin Neurol Neurosurg (2016) 144:53–8. doi: 10.1016/j.clineuro.2016.02.041 26971296

[188] Cormier S Leroy C Delezoide AL Silve C. Expression of fibroblast growth factors 18 and 23 during human embryonic and fetal development. Gene Expr Patterns (2005) 5:569–73. doi: 10.1016/j.modgep.2004.10.008 15749088

[189] Whitmore TE Maurer MF Sexson S Raymond F Conklin D Deisher TA. Assignment of fibroblast growth factor 18 (FGF18) to human chromosome 5q34 by use of radiation hybrid mapping and fluorescence *in situ* hybridization. Cytogenet Cell Genet (2000) 90:231–3. doi: 10.1159/000056775 11124520

[190] Reinhold MI Naski MC. Direct interactions of Runx2 and canonical wnt signaling induce FGF18. J Biol Chem (2007) 282:3653–63. doi: 10.1074/jbc.M608995200 17158875

[191] Katoh M Katoh M. FGF signaling network in the gastrointestinal tract (review). Int J Oncol (2006) 29:163–8. doi: 10.3892/ijo.29.1.163 16773196

[192] Lemmon MA Schlessinger J. Cell signaling by receptor tyrosine kinases. Cell (2010) 141:1117–34. doi: 10.1016/j.cell.2010.06.011 PMC2914105 20602996

[193] Schlessinger J Plotnikov AN Ibrahimi OA Eliseenkova AV Yeh BK Yayon A. Crystal structure of a ternary FGF-FGFR-heparin complex reveals a dual role for heparin in FGFR binding and dimerization. Mol Cell (2000) 6:743–50. doi: 10.1016/S1097-2765(00)00073-3 11030354

[194] Mohammadi M Olsen SK Ibrahimi OA. Structural basis for fibroblast growth factor receptor activation. Cytokine Growth Factor Rev (2005) 16:107–37. doi: 10.1016/j.cytogfr.2005.01.008 15863029

[195] Hou J Kan M Wang F Xu JM Nakahara M McBride G. Substitution of putative half-cystine residues in heparin-binding fibroblast growth factor receptors. loss of binding activity in both two and three loop isoforms. J Biol Chem (1992) 267:17804–8. doi: 10.1016/S0021-9258(19)37115-7 1325450

[196] Plotnikov AN Schlessinger J Hubbard SR Mohammadi M. Structural basis for FGF receptor dimerization and activation. Cell (1999) 98:641–50. doi: 10.1016/S0092-8674(00)80051-3 10490103

[197] Kelleher FC O’Sullivan H Smyth E McDermott R Viterbo A. Fibroblast growth factor receptors, developmental corruption and malignant disease. Carcinogenesis (2013) 34:2198–205. doi: 10.1093/carcin/bgt254 23880303

[198] Ornitz DM Itoh N. The fibroblast growth factor signaling pathway. Wiley Interdiscip Rev Dev Biol (2015) 4:215–66. doi: 10.1002/wdev.176 PMC4393358 25772309

[199] Xie X Guo P Yu H Wang Y Chen G. Ribosomal proteins: insight into molecular roles and functions in hepatocellular carcinoma. Oncogene (2018) 37:277–85. doi: 10.1038/onc.2017.343 28945227

[200] Li Q Kannan A DeMayo FJ Lydon JP Cooke PS Yamagishi H. The antiproliferative action of progesterone in uterine epithelium is mediated by Hand2. Science (2011) 331:912–6. doi: 10.1126/science.1197454 PMC3320855 21330545

[201] Joosten SPJ Spaargaren M Clevers H Pals ST. Hepatocyte growth factor/MET and CD44 in colorectal cancer: partners in tumorigenesis and therapy resistance. Biochim Biophys Acta Rev Cancer (2020) 1874:188437. doi: 10.1016/j.bbcan.2020.188437 32976979

[202] Pothuraju R Rachagani S Krishn SR Chaudhary S Nimmakayala RK Siddiqui JA. Molecular implications of MUC5AC-CD44 axis in colorectal cancer progression and chemoresistance. Mol Cancer (2020) 19:37. doi: 10.1186/s12943-020-01156-y 32098629 PMC7041280

[203] Yaghobi Z Movassaghpour A Talebi M Abdoli Shadbad M Hajiasgharzadeh K Pourvahdani S. The role of CD44 in cancer chemoresistance: a concise review. Eur J Pharmacol (2021) 903:174147. doi: 10.1016/j.ejphar.2021.174147 33961871

[204] Bugter JM Fenderico N Maurice MM. Mutations and mechanisms of WNT pathway tumour suppressors in cancer. Nat Rev Cancer (2021) 21:5–21. doi: 10.1038/s41568-020-00307-z 33097916

